# Mechanically Interlocked Double-Walled Covalent Organic Framework with Cucurbit[8]Uril-Stabilized Viologen Radical Dimers

**DOI:** 10.1021/jacs.6c01520

**Published:** 2026-07-23

**Authors:** Salma Abubakar, Bikash Garai, Nour Alkhatib, Rasha G. AbdulHalim, Thirumurugan Prakasam, Sudhir Sharma, Lydia Gkoura, Ribal Jabbour, Asif Equbal, Felipe Gándara, Jihan Dhainy, Devin Thrush, Claudia E. Avalos, Gobinda Das, Sharath Kandambeth, Na’il Saleh, Ramesh Jagannathan, Mark A. Olson, Mohamad El-Roz, Serdal Kirmizialtin, Mohamed Eddaoudi, Ali Trabolsi

**Affiliations:** † Chemistry Program, 167632 New York University Abu Dhabi, Saadiyat Island, Abu Dhabi 129188 PO Box, UAE; ‡ Water Research Centre, New York University Abu Dhabi, Saadiyat Island, Abu Dhabi 129118 PO Box, UAE; § Department of Chemistry and Chemical Biology, Indian Institute of Technology (Indian School of Mines) Dhanbad, Dhanbad 826004, Jharkhand, India; ∥ Engineering Division, New York University Abu Dhabi, Saadiyat Island, Abu Dhabi 129188 PO Box, UAE; ⊥ Center for Quantum and Topological Systems, New York University Abu Dhabi, Saadiyat Island, Abu Dhabi 129188 PO Box, UAE; # Materials Science Institute of Madrid, CSIC, Sor Juana Inés de la Cruz 3, Madrid 28049, Spain; ¶ Catalysis and Spectrochemistry Laboratory, CNRS, ENSICAEN, Caen University, Caen cedex 14050 PO Box, France; ∇ Department of Chemistry, New York University, New York 10003, New York, United States; ○ Physical Science and Engineering Division, 127355 King Abdullah University of Science and Technology, Thuwal 23955-6900, Saudi Arabia; ⧫ Department of Chemistry, College of Science, 11239 United Arab Emirates University, Al Ain 15551 PO Box, UAE; †† Department of Physical & Environmental Sciences, 14738 Texas A&M University Corpus Christi, Corpus Christi 78412, Texas, United States

## Abstract

The stabilization and functional integration of organic radicals within crystalline frameworks remain a significant challenge in materials chemistry, particularly for applications in optoelectronics and sensing. Herein, we report the design and synthesis of a radical-rich, polyrotaxanated covalent organic framework (VCB COF) featuring viologen-based radical cation dimers mechanically interlocked within cucurbit[8]­uril (CB[8]) macrocycles. This supramolecular architecture gives rise to a double-walled framework that significantly enhances radical stability, photophysical properties, and photoresponsivity. Electron paramagnetic resonance (EPR) analysis reveals that VCB retains over 72% of its radical signal after 7 days of air exposure, compared to just 22% in its nonrotaxanated analogue, TpV COF. Photophysical studies reveal a 3-fold increase in photoluminescence intensity, while photoelectrochemical measurements demonstrate a stable photocurrent density of 1.3 μA cm^–2^ under 405 nm illumination with reversible on/off switching. Molecular dynamics simulations together with solid-state NMR support the localization of CB[8] at COF junctions and are consistent with the formation of a double-walled architecture stabilized by dipolar and long-range noncovalent interactions. This work establishes a generalizable strategy for integrating persistent radicals within COFs through macrocycle-assisted encapsulation and mechanical bonding, opening new avenues for the development of robust photoactive, redox-active, and spin-functional materials.

## Graphical abstract

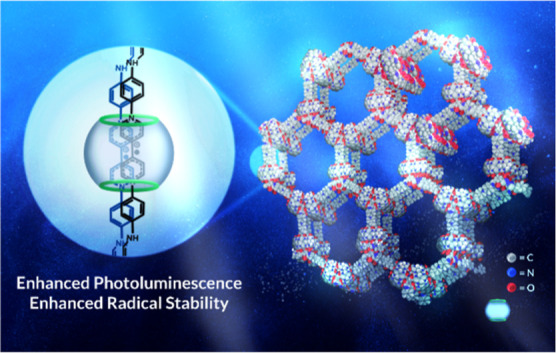

## 1 Introduction

Covalent organic frameworks (COFs) are a class of porous and reticular organic materials composed of light elements, valued for their structural tunability, controllable synthesis, permanent porosity, and high chemical and thermal stability.^1,2^ These properties have enabled their widespread application in areas such as sensing,^3^ gas storage,^4^ energy conversion,^5^ environmental remediation,^6,7^ bioimaging,^8,9^ and drug delivery.^10^ COFs incorporating organic radicals, molecular species containing one or more unpaired electrons, are particularly intriguing because of their unique physicochemical properties, such as enhanced electrical conductivity and magnetic behavior.^11–13^ Representative examples of organic radicals include triphenylamine radical cations,^14,15^ tetramethylpiperdinyl-1-oxyl (TEMPO),^16,17^ and viologen radical cations.^18,19^ Radical-containing COFs have shown unique physicochemical properties, enabling applications in photocatalysis,^20^ energy storage,^21^ and photothermal conversion.^22^ In addition, the photosensitivity of radical unit-based materials makes them promising candidates for photodetection.^23^ The practical use of organic radicals, however, hinges on their stability, especially under ambient conditions. Strategies to stabilize radicals include dimerization,^24,25^ steric hindrance,^24,26^ π-conjugation for charge delocalization,^27,28^ and encapsulation in mechanically interlocked architectures and host–guest systems.^29,30^

Viologens, or 4,4′-bipyridinium salts, are among the most studied redox-active molecules^31^ due to their ability to undergo two reversible one-electron reductions, switching between viologen dication (V^2+^), radical cation (V^•+^), and neutral (V^0^) states.^32–34^ These properties have enabled their use in electrochromic devices,^35^ organic redox-flow batteries,^36^ and supercapacitors.^37^ The radical cation form (V^•+^) is particularly notable for its tendency to pimerize^38^ in aqueous media, which is caused by the spin pairing and the accompanying radical–radical interactions.^19,39^ Additionally, viologens can form inclusion complexes with macrocycles such as calix­[n]­arenes and cucurbit­[n]­urils, with complexation behaviors dependent on both the viologen’s redox state and the macrocycle’s cavity size.^40–42^ Both radical pimerization and host-guest complexation have been shown to significantly enhance the stability of viologen radicals toward oxidation and other redox processes, which is advantageous for their practical applications.^43^

Cucurbit­[n]­urils (CB­[*n*], *n* = 5–8, or 10) are a family of glycouril-based macrocycles with hydrophobic cavities and identical carbonyl-fringed rims.^44^ They are widely known to form pseudorotaxanes by encapsulating guest molecules through a combination of the hydrophobic effect and ion–dipole interactions involving the carbonyl groups of CB­[n]’s.^45^ The incorporation of CB­[n]-based rotaxane units into materials often enhances their mechanical strength and thermal and chemical stability.^46,47^ Compared with its homologues CB[6] and CB[7], CB[8] possesses a larger cavity with an outer diameter of 17.5 Å and can simultaneously accommodate multiple guest molecules, forming 1:1:1 or 1:2 host–guest complexes.^48,49^ Notably, CB[8] binds strongly to viologens, with an association constant exceeding 10^5^ M^–1^ for the 1:1 V^2+^–CB­[8] inclusion complex.^50^ More importantly, CB[8] can simultaneously encapsulate two viologen radical cations, dramatically enhancing radical pimerization, with the association constant for viologen radical dimerization increasing by approximately 5 orders of magnitude.^50^ This unique capability has been exploited to construct stable radical-containing systems, including dendrimers and supramolecular organic frameworks (SOFs).^51,52^ However, reports of CB[8]-based COFs remain scarce,^53,54^ and the incorporation of CB[8]-stabilized viologen radical dimers into covalent organic frameworks has remained largely unexplored. More importantly, the ability of CB[8] to simultaneously host two viologen radical cations provides a unique opportunity to exploit radical dimerization as a structural motif within COFs, a feature that cannot be achieved using the smaller CB[7] homologue.

In this work, we present the synthesis of a radical-rich polyrotaxanated double-walled COF (VCB), constructed from 2,4,6-triformylphloroglucinol (Tp) and CB[8]-encapsulated viologen radical-cation dimers. Exploiting the ability of CB[8] to simultaneously host two viologen radical cations, a [3]­pseudorotaxane precursor is first formed and subsequently incorporated into a β-ketoenamine-linked framework through condensation with Tp. The resulting architecture differs fundamentally from previously reported CB[7]-based polyrotaxanated COFs,^55^ as the larger CB[8] cavity promotes viologen radical dimerization and enables the formation of a mechanically interlocked double-walled architecture. As a consequence, VCB exhibits enhanced radical stability, improved photophysical properties, and stable photoelectrochemical performance. Compared with the nonrotaxanated analogue TpV,^55^ VCB displays a stable photocurrent density of 1.3 μA cm^–2^, significantly enhanced photoluminescence, and remarkable resistance toward radical degradation under ambient conditions. The electron paramagnetic resonance (EPR) signal intensity decreases by only 28% after 7 days of air exposure, whereas TpV loses approximately 78% of its signal over the same period. These results show that CB[8]-mediated radical dimer stabilization and mechanical interlocking provide an effective strategy for integrating persistent radicals into crystalline framework materials.

## 2 Results and Discussion

### 2.1 Formation and Characterization of the CB[8]⊃(V^•+^)_2_ Pseudorotaxane

Viologen radical cations (V^•+^) were first generated by reducing an aqueous solution of the viologen dication (V^2+^) using sodium dithionite (Na_2_S_2_O_4_, SDT). The formation of V^•+^ was confirmed by EPR spectroscopy, which displayed a strong signal with a *g*-factor of 2.012, close to the free electron value of 2.0045, characteristic of organic radicals (Figure S1).^56,57^ Successful reduction of V^2+^ was further supported by ultraviolet–visible (UV–vis) absorption spectroscopy. The disappearance of the characteristic UV absorption band for the dicationic compound at 255 nm and the appearance of a new band at 317 nm following SDT treatment indicated formation of the radical species (Figure S2).^51,58^ Exploiting the ability of CB[8] to simultaneously accommodate two guests, a 1:2 inclusion complex was formed between CB[8] and the viologen radical cations, yielding the [3]­pseudorotaxane, CB[8]⊃(V^•+^)_2_ (Figure 1). EPR analysis confirmed the persistence of radical species in the resulting CB[8]⊃(V^•+^)_2_ (Figure S1). The formation of the 1:2 complex was further confirmed by UV–vis absorption spectroscopy, which revealed a new broad adsorption bands centered at 468 and 575 nm (Figure S2). This band is blue-shifted from ∼600 nm band typical of monomeric viologen radical cations, suggesting a successful formation of the 1:2 inclusion complex.^59^ In addition, a broad near-infrared (NIR) band centered at 981 nm was observed and assigned to viologen radical dimer (pimer) formation.^60,61^ The emergence of these new visible and NIR absorption features is consistent with radical–radical interactions promoted by CB[8] encapsulation.

**Figure 1:**
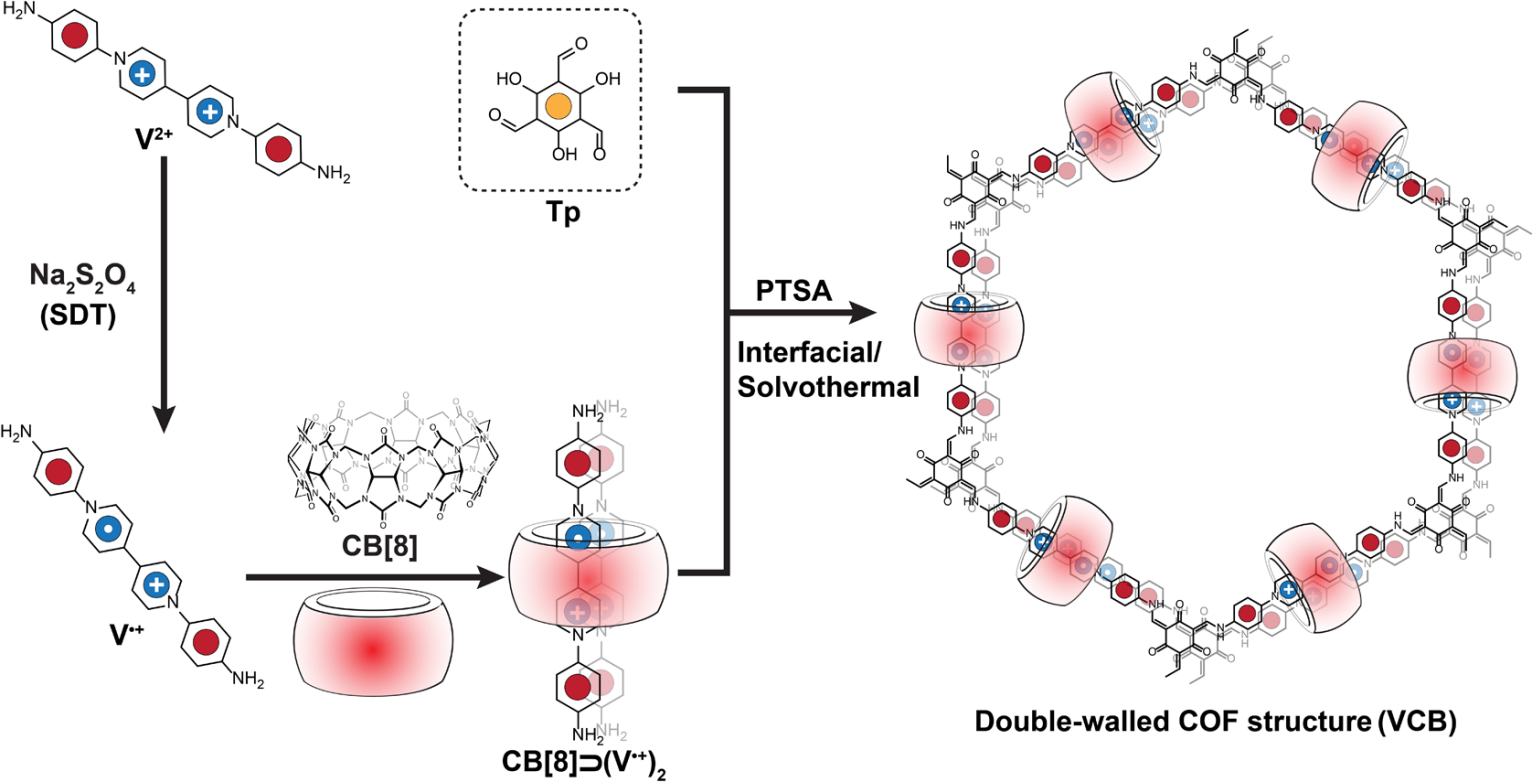
Schematic illustration of the synthesis of VCB COF. The bipyridinium precursor is first reduced with sodium dithionite, followed by complexation with cucurbit[8]­uril to form a [3]­pseudorotaxane, CB[8]⊃(V^•+^)_2_. Subsequent Schiff-base condensation with 2,4,6-triformylphloroglucinol yields a double-walled, polyrotaxanated COF (VCB COF). The counteranions have been eliminated for clarity of the structure.

## 3 Synthesis and Characterization of VCB COF

The polyrotaxanated COF was synthesized using two distinct methods. The first approach employed the liquid–liquid interfacial polymerization strategy (Figure 1). In this method, an aqueous solution of CB[8]⊃(V^•+^)_2_ and *p*-toluenesulfonic acid (PTSA) catalyst was carefully layered over a biphasic water–dichloromethane system (Figure S3). The dichloromethane layer contained Tp, which serves as the tritopic node of the framework. This interfacial setup slows the diffusion of the reactive components, enabling controlled framework growth through Schiff-base condensation.^62^ The reaction was carried out under inert conditions, resulting in the formation of a thin, dark film at the water–organic interface. For comparison, VCB was also synthesized under solvothermal conditions using aqueous DMAc as the reaction medium. The resulting material exhibited improved crystallinity and was therefore used for subsequent characterization studies.

Fourier-transform infrared (FT-IR) spectroscopy supported the successful condensation reaction. The characteristic stretching bands corresponding to the primary amines (–NH_2_, 3181 and 3308 cm^–1^) and aldehyde carbonyl groups (C=O, 1635 cm^–1^) of the starting materials V^2+^ and Tp, respectively, disappeared for the product (Figure S4). The loss of these bands indicates efficient consumption of the precursor functional groups during condensation. In addition, the appearance of a C=C stretching band at 1577 cm^–1^ is consistent with the formation of the β-ketoenamine linkage resulting from irreversible enol–keto tautomerization. Notably, the carbonyl stretching bands of CB[8] shift from 1712 cm^–1^ to 1722 cm^–1^ upon framework formation, providing further evidence for the incorporation of CB[8] within the VCB framework.^55,63^

Evidence for network formation and framework composition was obtained through ^1^H–^13^C cross-polarization magic angle spinning (CP-MAS) solid-state NMR spectroscopy (600 MHz). VCB exhibited resonances at approximately 54, 73, and 157 ppm (Figure 2a), which are assigned to –CH_2_, –CH, and –C=O groups of the CB[8] macrocycle, respectively.^64^ These resonances are broader than those of the pure CB[8] (Figure S5), which can be attributed to shortened T_2_ relaxation times arising from interactions between the CB[8] macrocycles and the viologen units, consistent with host–guest complex formation.^65^ Additional resonances at ∼114 and ∼149 ppm, corresponding to exocyclic C=C and carbon atoms bonded to the –NH group, respectively, are consistent with formation of the β-ketoenamine linkage through irreversible tautomerism.^66,67^ Given the inherently low sensitivity of conventional solid-state NMR due to anisotropic interactions,^68^ dynamic nuclear polarization (DNP) was employed to enhance signal intensity.^69^ VCB was therefore analyzed using DNP-enhanced ^1^H–^13^C CP-MAS NMR spectroscopy at 100 K and 600 MHz (Figure 2b).^70^ For these measurements, the sample was impregnated with a 10 mM AMUPOL (for CP-MAS) or ASYMPol-Pok (for direct excitation using single pulse and subsequent detection) biradical polarizing agent in a 60:30:10 mixture of *d*_8_-glycerol:D_2_O/H_2_O, as detailed in the Supporting Iinformation.^71^ The DNP-enhanced spectrum showed a pronounced resonance at ∼184 ppm, attributed to the keto carbon atoms, providing additional evidence for successful framework formation through Schiff-base condensation followed by keto–enamine tautomerization.

**Figure 2:**
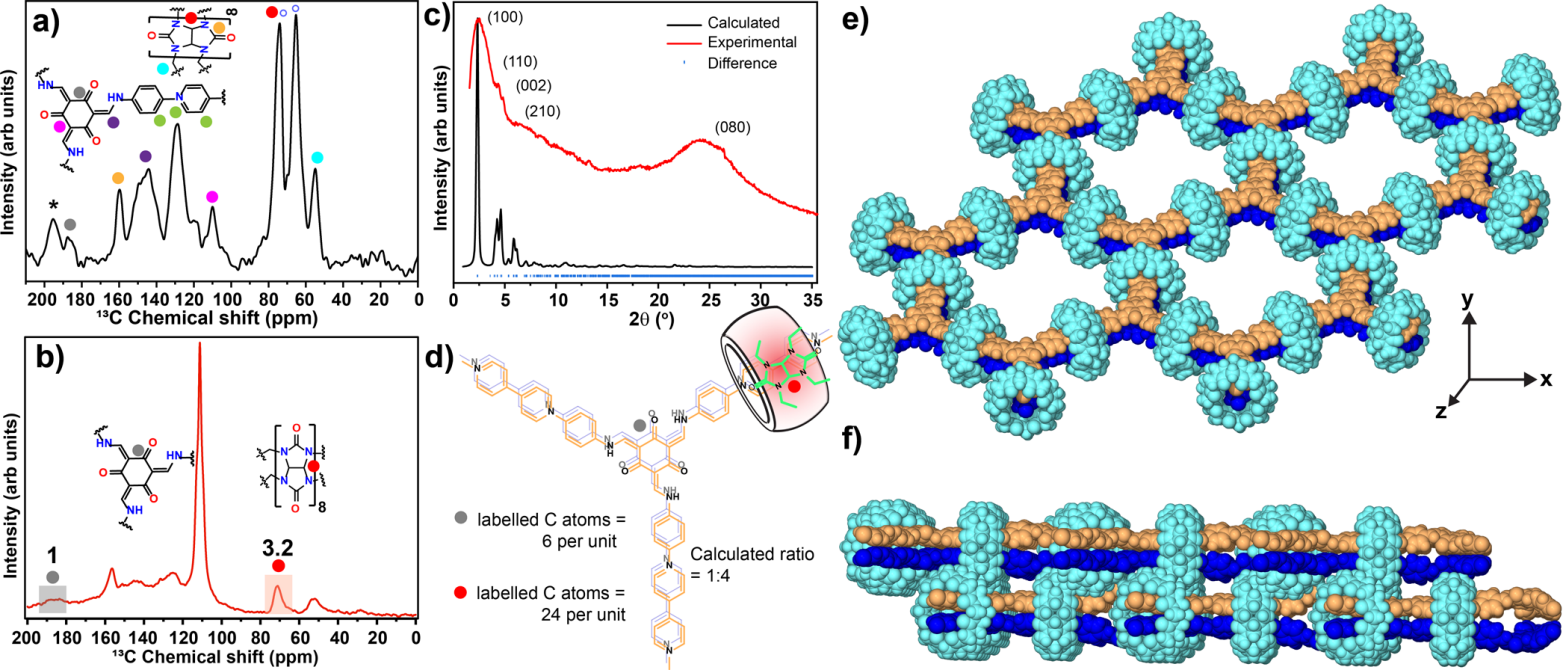
(a) Solid state ^13^C CP-MAS DNP NMR spectra highlighting characteristic signals: carbonyl carbons of the framework (gray), carbonyls from CB[8] (orange), exocyclic C=C (pink), –CH (purple), –CH (red), and –CH_2_ (cyan) of the macrocycle. The peak labeled with * corresponds to a spinning sideband and the peak labeled with open circle originates from glycerol. (b) Solid-state DNP-enhanced ^13^C direct excitation NMR spectrum of VCB COF (600 MHz, 100 K) showing enhanced signals corresponding to the same assignments. (c) PXRD pattern of VCB COF: comparison of experimental data (red) and simulated profile (black). (d) Location of representative C atoms in the structure used for calculating the stoichiometric ratio and the theoretical ratio. (e) Top view and (f) side view of the proposed model structure of VCB COF, showing the proposed hexagonal arrangement and double-walled architecture with CB­[8] units interlocking the layers.

The intensity ratio of the –CH to –CH_2_ resonances in the DNP direct-excitation spectrum was lower than that observed in the CP-MAS spectrum because of partial overlap with glycerol resonances appearing at 63 and 73 ppm.^70^ Since ^1^H–^13^C CP-MAS is not inherently quantitative due to variable cross-polarization transfer efficiencies, quantitative analysis was performed using direct-excitation ^13^C MAS NMR with efficient heteronuclear decoupling and a long recycle delay (20 s) to minimize relaxation-related intensity differences (Figure 2b). Owing to the exceptional chemical stability of the β-ketoenamine-linked framework, chemical digestion approaches commonly used for compositional analysis of COFs were not feasible. Instead, the integrated peak areas corresponding to the framework carbonyl carbons (–C=O) and the CB[8] methine carbons (–CH) were used to estimate the number of CB[8] macrocycles per viologen unit. Each CB[8] macrocycle contains 16 methine carbons and accommodates a viologen radical dimer, thereby mechanically interlocking two framework layers, whereas each Tp unit contributes three carbonyl (–C=O) carbons at the central trigonal nodes. Based on the proposed [3]­rotaxane architecture, the theoretical –CH:–C=O peak-area ratio is therefore 1:4 (Figure 2d). Experimentally, a ratio of approximately 1:3.2 was obtained from the direct-excitation spectrum (Figure 2b). Considering the peak broadening and partial overlap with glycerol resonances, this level of agreement is considered reasonable for a direct-excitation solid-state NMR experiment. The observed ratio therefore provides strong support for the proposed double-walled polyrotaxanated COF architecture.

Powder X-ray diffraction (PXRD) analysis revealed broad reflections at both low and high angles, consistent with the formation of an ordered layered structure possessing periodic in-plane organization (Figure 2c). The most intense reflection is observed at 2θ = 2.4°, accompanied by weaker reflections at 4.3° and 4.8°, as well as a broad feature centered at 26.4°, consistent with an ordered layered structure possessing a large in-plane lattice. Based on the geometry of the building blocks, an *hcb* topology was proposed. Structural models were constructed using Materials Studio, and optimized with guidance from the experimental PXRD data. The resulting model incorporates rotaxanated moieties within the hexagonal pores and is consistent with a mechanically interlocked double-walled architecture (Figure 2e,f). The geometrically optimized structure belongs to the monoclinic *Pm* space group, with the layers extending parallel to the ac plane. Two sets of rotaxanated layers are present within each unit cell and adopt a staggered arrangement that optimizes packing of the CB[8] macrocycles. Within this model, the reflection at 2θ = 2.4° is assigned to the overlap of the (100) and (101̅) reflections (calculated 2θ = 2.30° and 2.34°, respectively), whereas the reflections at 4.3° and 4.8° correspond to the (011) and (200) planes (calculated 2θ = 4.25° and 4.59°, respectively). The broad feature centered at 26.4° is attributed to limited stacking coherence and structural disorder along the layer-stacking direction. The wider aperture of CB[8] (portal diameter of 8.8 Å, Figure 3a) can simultaneously accommodate two viologen radical cations separated by approximately 3.8 Å, facilitating the formation of a radical π-dimer. Encapsulation of the viologen radical dimer within the CB[8] cavity yields the [3]­pseudorotaxane CB[8]⊃(V^•+^)_2_. Subsequent reaction of the terminal amine groups on the viologen linkers with the aldehyde functionalities of Tp results in the formation of β-ketoenamine linkages and the formation of covalent two-dimensional (2D) network layers. According to the proposed structural model, each CB[8] macrocycle links two neighboring viologen units from adjacent layers, thereby giving rise to a mechanically interlocked architecture consistent with a double-walled framework. Lateral growth of these mechanically interlocked layers produces extended 2D VCB nanosheets, which further assemble through an offset AB-type stacking arrangement (Figure 3b). Additional structural characterization was conducted to probe the nitrogen environment and provide further insight into the spatial arrangement of the macrocycles and linkers within the framework. Although direct observation of the ^15^N nuclei is challenging because of their low natural abundance (0.37%) and gyromagnetic ratio,^72^ DNP-enhanced ^1^H–^15^N CP spectra were successfully acquired at 100 K (Figure S6). The spectrum revealed three distinct resonances at 218, 108, and 68 ppm. The signal at 108 ppm was assigned to the tertiary amines of the CB[8] macrocycles, whereas the signal at 68 ppm corresponds to the secondary amines of the VCB backbone, consistent with reported chemical shift ranges for secondary aliphatic amines.^73,74^ The resonance at 218 ppm was assigned to the iminium species. Although iminium nitrogen atoms typically resonate at a higher frequency, often approaching 300 ppm,^75^ the observed upfield shift suggests enhanced electronic shielding, possibly arising from encapsulation within the CB[8] cavity. Interestingly, the DNP enhancement was more pronounced for the ^15^N nuclei within the CB[8] macrocycle, suggesting greater accessibility of these sites to the polarizing solution relative to the more embedded nitrogen atoms of the framework backbone.^76^ In contrast, the lower enhancement observed for the backbone nitrogen atoms may additionally reflect paramagnetic relaxation effects arising from the unpaired electrons present in the radical-containing framework, which are known to attenuate signal intensities in solid-state NMR experiments.^77^ Collectively, the PXRD, solid-state NMR, and DNP-enhanced spectroscopic analyses provide evidence consistent with the proposed mechanically interlocked double-walled architecture of VCB.

**Figure 3:**
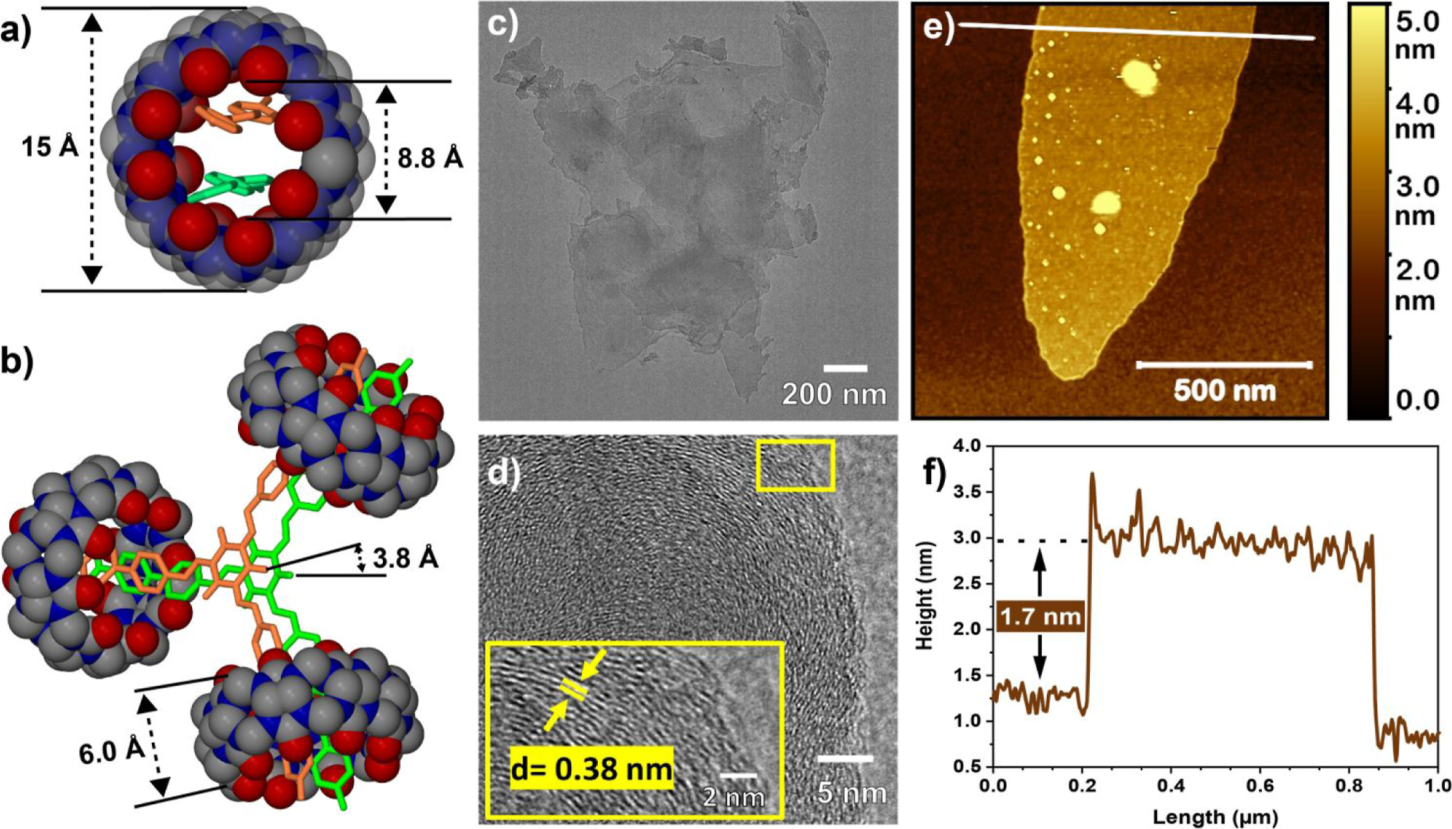
Structural and morphological characterization of VCB COF. (a) Space-filling model showing a CB[8] macrocycle encapsulating two viologen units in a [3]­pseudorotaxane geometry. (b) Molecular model illustrating the formation of the polyrotaxanated COF, where viologen dimers are bridged by trialdehyde (Tp) linkers and threaded through CB[8] macrocycles, forming the double-walled architecture. (c) HR-TEM image revealing stacked COF nanosheets. (d) HR-TEM highlighting lattice fringes with an interlayer *d*-spacing of ∼0.38 nm, consistent with π–π stacking between interlocked layers. (e) AFM topographic image confirming the presence of nanosheets. (f) Height profile extracted from the AFM image in (e), indicating a step height of 1.7 ± 0.2 nm, which corresponds to the outer diameter of the CB[8] macrocycle and is consistent with the proposed mechanically interlocked double-walled architecture of VCB..

The morphology of the polyrotaxanated COF was examined by scanning electron microscopy (SEM), high-resolution transmission electron microscopy (HR-TEM), and atomic force microscopy (AFM). SEM images showed a 2D sheet-like morphology (Figure S7), which was corroborated by HR-TEM observations showing stacked, thin COF layers (Figure 3c). At higher magnification, HR-TEM images revealed clear lattice fringes with an average *d*-spacing of approximately 0.38 nm, consistent with π–π stacking interactions between adjacent framework layers (Figure 3d). AFM analysis further confirmed the formation of thin nanosheets, with height profiles (Figure 3e,f) indicating a thickness of 1.7 ± 0.2 nm. This value is comparable to the outer diameter of CB[8] and is therefore consistent with the proposed mechanically interlocked double-walled architecture.^48^

UV–vis–NIR diffuse reflectance spectroscopy (DRS) of VCB revealed a broad absorption band extending from the UV into the visible region (Figure S8). An absorption edge was observed at ∼560 nm, which is attributed to the viologen radical cations embedded within the framework.^78,79^ In comparison, the nonrotaxanated COF, TpV, exhibited an absorption edge at a longer wavelength (∼670 nm), highlighting the influence of CB[8] incorporation on the electronic structure of the material.

The thermal stability of VCB was evaluated using thermogravimetric analysis (TGA). The initial weight-loss of 10% observed below 100 °C corresponds to the evaporation of adsorbed moisture (Figure S9). VCB remained thermally stable up to approximately 350 °C, with the major decomposition occurring between 350 and 440 °C, corresponding to degradation of the framework and formation of volatile decomposition products.^80,81^ Notably, this thermal stability surpasses that of TpV, which begins to decompose above 201 °C.^55^ The enhanced thermal robustness of VCB is consistent with the additional structural reinforcement provided by the mechanically interlocked architecture and the incorporation of CB[8] macrocycles.

To gain deeper insight into the structural behavior of the VCB COF, molecular dynamics (MD) simulations were performed. The initial simulation setup included a rotaxanated COF framework with chloride counterions (Figure S10a). A series of 100 ns MD simulations were conducted for systems incorporating varying numbers of CB[8] macrocycles (*n* = 2 to 12) to evaluate the structural dynamics and energetic stability as a function of macrocycle density (Figure S10b). Representative equilibrium snapshots (Figure 4a,b) from the MD simulations revealed significant positional rearrangements of the CB[8] units relative to their initial locations. To systematically study the changes of the macrocycle positions, we tracked the spatial distance of each macrocycle from a junction point, with the shortest distance from the center of mass of the macrocycle to the junction point during the simulations. The analysis demonstrated that, as the number of CB[8] macrocycles increases, the macrocycles progressively localize at the junction points of the COF framework. To investigate the origin of this localization behavior, we examined the orientation of the macrocycle dipole moments. The analysis showed that the dipole moments of the CB[8] units align with the COF junctions (Figure 4c), suggesting that dipolar interactions contribute to the preferential localization of the macrocycles. To quantify the energetic effect of macrocycle incorporation, we calculated the interaction energy change (Δ*U*_int_) associated with different macrocycle loadings within the framework. As shown in Figure 4d, Δ*U*_int_ decreases with increasing macrocycle content, reaching a minimum when all viologen linkers are encapsulated by CB[8] (i.e., *n* = 12). This energetically favorable state is consistent with the proposed fully interlocked structure of VCB.

**Figure 4:**
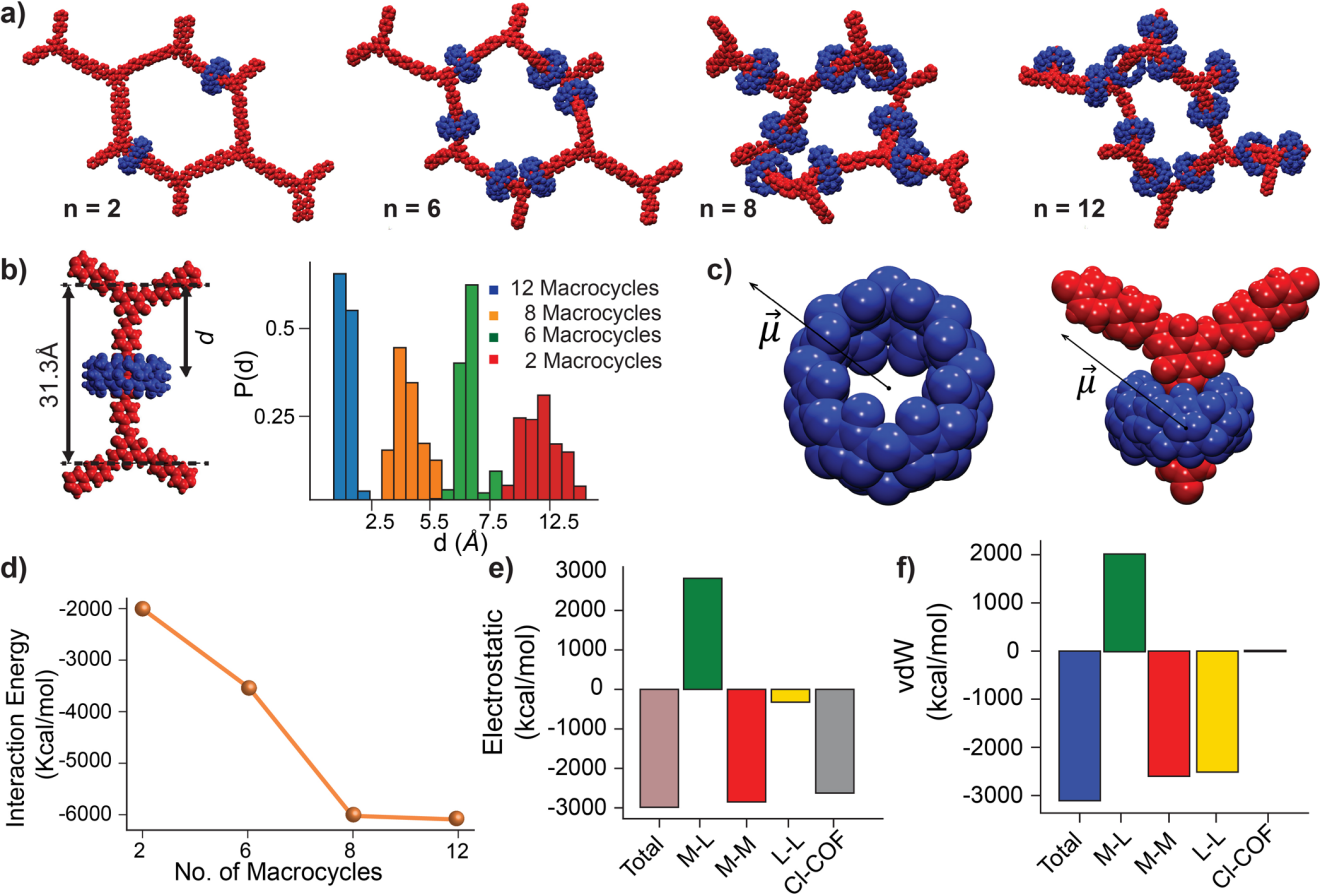
Structural and energetic analysis of VCB. (a) Snapshots of four distinct COF systems containing 2, 6, 8, and 12 macrocycles at equilibrium. (b) A schematic illustrating the measured distance between linker and macrocycle to monitor macrocycle positions, in addition to the probability distribution of macrocycle positions in each system. (c) Computed dipole moment of the macrocycle. (d) Interaction energy of COFs with varying numbers of macrocycles. (e,f) The partition of interaction energy to electrostatic and van der Waals terms for the lowest energy state (*n* = 12): all energy pairs (Total), energy between macrocycle and linker (M-L), between macrocycles (M–M), between linkers (L–L), and between chloride ions and the entire COF (Cl–COF).

To further evaluate alternative thermodynamically accessible configurations, density functional theory (DFT) calculations were performed on a series of plausible structural intermediates and candidate architectures (Figure S11). Specifically, we considered: (i) a noncomplexed double-layer framework with a free macrocycle (DL + M), (ii) a single-layer framework containing a bound macrocycle together with a second isolated layer (SLM + SL), and (iii) a fully interlocked double-layer framework containing the macrocycle (DLM). All energies were referenced to a system consisting of two isolated single-layer frameworks and one free macrocycle (SL + SL + M). The calculations reveal a clear stabilization trend upon macrocycle incorporation. While the noncomplexed double-layer structure (DL + M) exhibits only moderate stabilization relative to the reference state, incorporation of the macrocycle into the framework (SLM + SL) results in a substantial decrease in energy. The fully interlocked double-layer structure (DLM) was found to be the lowest-energy configuration among all structures examined (Δ*E* ≈ – 34 eV). These results indicate that macrocycle incorporation is the dominant stabilizing factor, while formation of the double-layer arrangement provides an additional energetic benefit. Among the structures considered, the fully interlocked double-layer architecture is thermodynamically the most favorable, providing additional thermodynamic support for the proposed double-walled, mechanically interlocked VCB architecture.

To elucidate the mechanism of stability achieved by integrated macrocycles, we partitioned the lowest energy state (*n* = 12) into van der Waals and Coulomb contributions (Figure 4e,f). In addition, each energy term was decomposed into pairwise interactions between macrocycle and linker (M-L), Linker–Linker (L–L), Macrocycle–Macrocycle (M–M), and chloride interactions with the remainder of the framework (Cl–COF). Interestingly, contributions from each energy term suggest that both van der Waals and Coulomb energy terms contribute almost equally to the overall stabilization. While M-L interactions are generally unfavorable, the VCB framework is predominantly stabilized by long-range macrocycle–macrocycle (M–M) interactions, which promote localization of the CB[8] units at the framework junctions. To further probe the thermal stability of the framework, quantum-based molecular dynamics (QMD) simulations were performed on a representative VCB structure. The simulations indicated that bond-breaking events occur preferentially within the COF backbone, while the CB[8] macrocycle remains comparatively stable. At elevated temperatures, disruption of the COF network precedes decomposition of the CB[8] macrocycle, consistent with the experimentally observed thermal behavior (Figure S12).

The porosity and surface characteristics of VCB were investigated by nitrogen sorption analysis at 77 K. The isotherm exhibits a Type II profile (Figure S13a), indicating nonporous or macroporous character. The Brunauer–Emmett–Teller (BET) surface area and pore volume of VCB were measured to be 31.5 m^2^·g^–1^ and 0.15 cm^3^·g^–1^, respectively. For comparison, the non rotaxanated analogue TpV has a BET surface area of 106 m^2^·g^–1^, whereas rotaxanation with CB[7] in TpVCB[7] reduces the surface area to approximately 20 m^2^·g^–1^.^55^ The relatively low surface area of VCB can be attributed to several structural factors, including (i) the presence of bulky CB[8] macrocycles within the framework, (ii) restricted pore accessibility arising from the mechanically interlocked double-walled architecture and offset layer stacking, and (iii) partial occupation of the pore volume by chloride counterions.^82^ These combined effects reduce nitrogen uptake and consequently lower the measured BET surface area relative to conventional COFs. Pore-size analysis revealed a characteristic pore diameter of approximately 1.7 nm (Figure S13b), which is in good agreement with the effective pore aperture of ∼1.8 nm predicted from the structural model after accounting for the offset stacking arrangement of the framework layers. This agreement further supports the proposed structural model.

The mechanical properties of VCB nanosheets were investigated using AFM-based techniques, namely force versus distance (F/D) spectroscopy and nanoindentation. Representative nanoindentation and F/D curves are shown in Figure 5a,b. Prior to mechanical testing, a preliminary AFM topographic scan was performed, and five distinct positions on an individual nanosheet were selected for measurements (Figure S14). These predefined locations were then used to acquire the F/D and nanoindentation data (Figure 5a). The F/D measurements revealed that the VCB nanosheets exhibit an average adhesion energy of 130 ± 10 fJ, which is higher than that of our previously reported TpV nanosheets.^55^ This suggests an increased work of adhesion in VCB, likely arising from the mechanically interlocked structure and the presence of the CB[8] macrocycles. Additionally, the measured pull-off force was 8.7 ± 0.5 nN, consistent with strong interfacial adhesion.^83^

**Figure 5:**
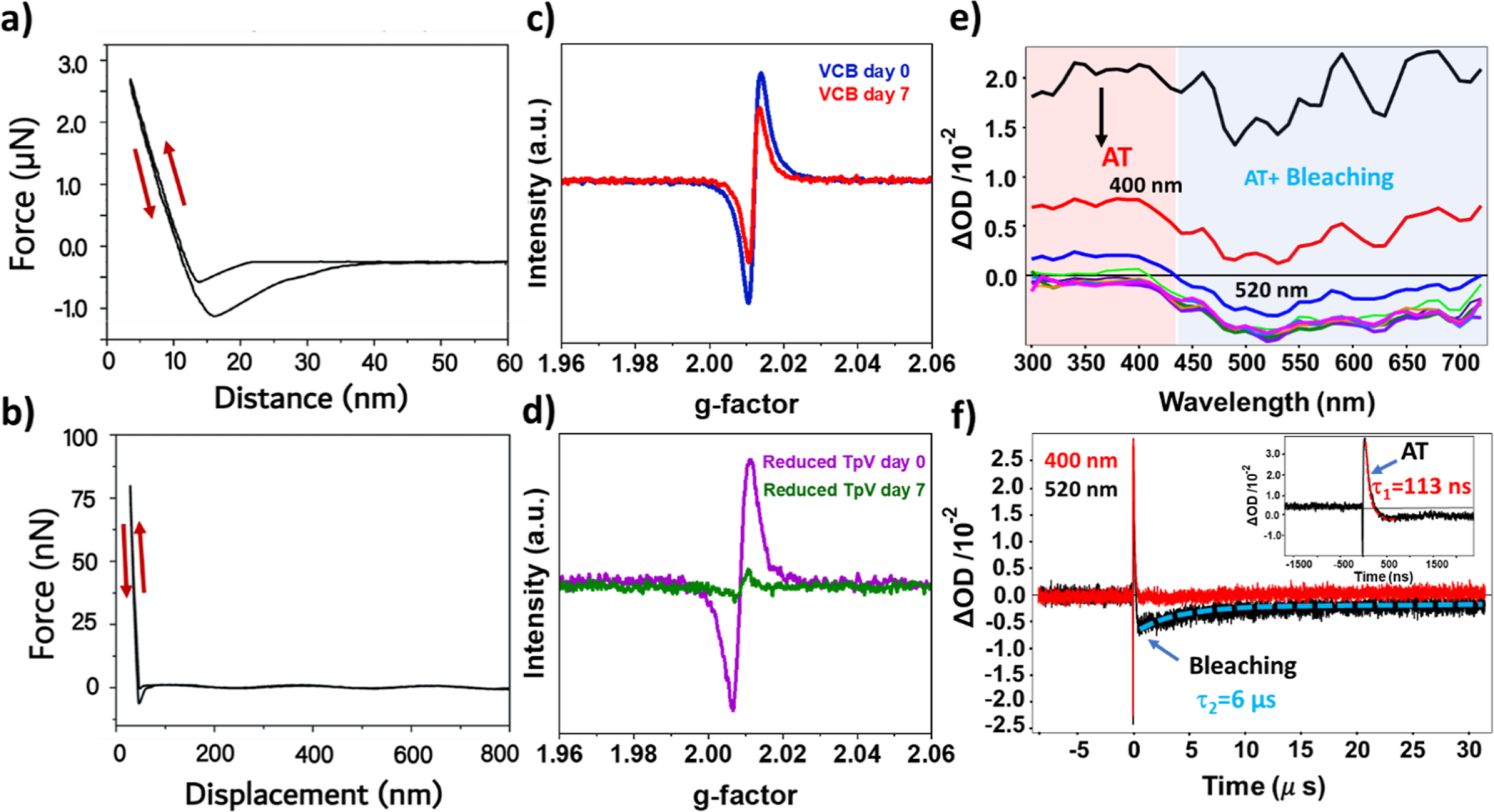
Mechanical characterization of VCB film showing (a) AFM nanoindentation plot, and (b) a typical force versus distance (F/D) curve. EPR spectra of (c) VCB and (d) TpV COF recorded immediately after treatment with SDT (day 0) and after 7 days of exposure to air. The VCB sample retains most of its radical signal over time, indicating high air-stability of the viologen radical cations. In contrast, TpV shows significant signal quenching, highlighting the superior radical stabilization afforded by the polyrotaxanated architecture of VCB. (e) Time-resolved absorption transient (AT) spectra of VCB COF in DMSO following excitation at 355 nm. Each trace represents 100 ns time slices, highlighting a broad AT band (300–700 nm) and a distinct bleaching signal centered at 520 nm (blue zone). (f) Kinetic decay profiles of the transient absorption at 400 nm (red) and bleaching at 520 nm (blue), showing lifetimes of ∼113 ns (τ_1_) and ∼6 μs (τ_2_), respectively. Inset: zoom-in of the bleaching signal at 520 nm, indicating long-lived photoinduced species.

Mechanical stiffness was assessed via AFM nanoindentation from which Young’s modulus and microhardness values of 4.2 ± 0.3 GPa and 5.3 ± 0.5 MPa, respectively were obtained.^84^ Both values are higher than those reported for TpV^55^ (Table S1), indicating enhanced mechanical robustness. These results suggest that the polyrotaxanated CB[8]-based architecture contributes to the improved mechanical properties of VCB relative to its nonrotaxanated TpV analogue.

## 4 Radical Stability

The stability of the viologen radical species within the VCB COF was investigated using EPR spectroscopy. As shown in Figure 5c,d, the EPR spectra confirm the presence of radical species within the framework. Previous studies have reported that viologen-based radicals exhibit a degree of air-stability as a result of radical pimerization.^85^ However, relatively little is known about the long-term stability of such radical dimers in the solid state, particularly following encapsulation within macrocyclic hosts. To evaluate the radical content of the framework, VCB was treated with sodiumdithionite (SDT) under reducing conditions. Notably, the EPR signal intensity remained largely unchanged following SDT treatment (Figure S15), indicating that a substantial fraction of the viologen units was already present in the radical state prior to reduction. Under ambient conditions, the polyrotaxanated VCB framework exhibited remarkable radical stability, retaining approximately 72% of its EPR signal intensity after 7 days of air exposure (Figure S16). In contrast, the reduced nonrotaxanated TpV COF lost approximately 78% of its signal over the same period. These observations are consistent with enhanced radical stabilization arising from the synergetic effects of viologen radical pimerization and CB[8]-mediated encapsulation. The mechanically interlocked architecture likely protects the radical sites from oxidative degradation, resulting in substantially improved radical persistence relative to nonrotaxanated analogues.

To distinguish between true mechanical interlocking and simple pore adsorption of CB[8], a series of control experiments were performed under inert glovebox conditions. First, the nonrotaxanated TpV COF was synthesized and chemically reduced with SDT to generate viologen radical species. Radical formation was confirmed by EPR spectroscopy (Figure S17, black trace). The reduced TpV material was then exposed to a CB[8] solution for 3 days to evaluate whether adsorption of the macrocycle within the pores could reproduce the radical stabilization observed in VCB. After only 1 h, EPR analysis of the CB[8]-adsorbed reduced TpV exhibited a significant decrease in EPR signal intensity (Figure S17, green trace). After 3 days, further signal attenuation was observed (Figure S17, purple trace), despite successful adsorption of CB[8] into the material. Adsorption of CB[8] was confirmed by FT-IR spectroscopy through the appearance of the characteristic carbonyl stretching band at 1732 cm^–1^ (Figure S18, green trace), consistent with the corresponding feature observed for VCB (Figure S18, maroon trace). Upon subsequent exposure of the CB[8]-adsorbed TpV sample to air, the EPR signal became nearly negligible (Figure S17, orange trace), closely resembling the behavior of the nonrotaxanated TpV framework. These results indicate that simple pore adsorption of CB[8] is insufficient to provide meaningful radical stabilization. In contrast, the significantly enhanced radical persistence observed for VCB is consistent with stabilization arising from the mechanically interlocked architecture generated through CB[8]-mediated rotaxanation.

### 4.1 Photoelectrochemical Properties

CB­[8] encapsulation modifies the electronic environment of the viologen radical dimers, as evidenced by the changes observed in the UV–vis absorption spectra (Figure S2). These spectral changes are consistent with enhanced radical–radical interactions, increased electronic delocalization, and stronger π–π interactions within the encapsulated system. These effects are expected to influence the excited-state properties of the framework and may contribute to the improved photoelectrochemical performance observed for VCB. The photoresponsive behavior of the VCB COF was evaluated by measuring its photocurrent density under illumination. At 0 V, the material showed negligible response. However, upon applying a +1 V bias and exposing the sample to 405 nm LED light, a photocurrent of ∼1.3 μA cm^–2^ was recorded (Figure S19). Under alternating light/dark cycles, the photocurrent remained nearly constant over at least three cycles, indicating reversible behavior. The minor decrease observed across cycles is likely due to slight dissolution of the COF/Nafion film by the NH_4_Cl electrolyte (0.1 M). These results highlight the potential of the polyrotaxanated VCB COF for photoresponsive and optoelectronic applications.

Photophysical properties were further investigated using nanosecond time-resolved photoluminescence (TR-PL) spectroscopy in a DMSO suspension. Compared to the reference TpV COF, VCB exhibited more than a 3-fold increase in photoluminescence intensity, with an emission maximum at 480 nm upon excitation at 355 nm (Figure S20). The photoluminescence lifetime was below the instrument resolution (<10 ns), consistent with fluorescence and the absence of long-lived phosphorescent states. This enhanced luminescence is consistent with CB[8]-mediated stabilization of viologen radical cation dimers and the modified electronic environment created by CB[8] encapsulation.^86^ Transient absorption spectroscopy (TAS) provided additional insight into the excited-state dynamics. Upon excitation at 355 nm, VCB displayed a broad transient absorption (TA) band spanning 300–700 nm, along with a distinct bleaching band centered at 520 nm (Figure 5e). Similar but less intense signals were observed under 532 nm excitation. Kinetic analysis revealed that the transient absorption decayed with a lifetime of ∼113 ns, while the bleaching band exhibited a longer lifetime of ∼6 μs (Figure 5f). Concerning the long-lived bleaching feature, we assign this signal to the formation of a transient photoinduced state associated with the viologen radical dimers, which exhibits reduced absorbance in the spectral region corresponding to the ground-state absorption of CB[8]⊃(V^•+^)_2_. The oxygen-insensitive nature of the transient signal rules out the involvement of triplet excited states and is instead consistent with a radical-associated excited state or a charge/radical transfer process within the framework.

Notably, the excited states were insensitive to oxygen, ruling out triplet-state involvement. The observed transient features are consistent with excitation of the viologen radical cations followed by nonradiative relaxation processes. The bleaching band is consistent with the formation of a transient photoinduced state exhibiting lower absorbance than the ground-state VCB framework and may arise from a charge- or radical-transfer process. The transient decrease in absorbance observed in the 450–700 nm region (highlighted in blue in Figure 5e) is consistent with this interpretation. The presence and remarkable stability of the viologen radical species are independently supported by the EPR measurements, while the transient absorption data demonstrate that these radical-containing states can participate in relatively long-lived photoinduced processes. Importantly, the absence of persistent species postexcitation and the oxygen insensitivity underscore the photostability of VCB under ambient conditions. It should be noted that a direct comparison between the time scales obtained from transient absorption spectroscopy and EPR measurements is not possible because the two techniques probe different physical processes under different experimental conditions. In addition, the TAS measurements were performed in a DMSO suspension, whereas the EPR measurements were conducted on solid samples. Differences in the local environment and relaxation dynamics can therefore lead to substantially different observed time scales.

## 5 Conclusion

In summary, we have synthesized and characterized a redox-active, mechanically interlocked covalent organic framework (VCB), designed through the pre-encapsulation of viologen radical cation dimers within CB[8] macrocycles, followed by liquid–liquid interfacial Schiff-base polymerization with a tritopic trialdehyde. The proposed double-walled architecture is supported by direct solid-state ^13^C MAS NMR spectroscopy, which revealed a CB[8]-to-viologen ratio consistent with the proposed structural model, as well as by UV–vis and EPR spectroscopy, which provide evidence for the presence of stable viologen dimers within the framework.

The integration of CB[8] not only conferred mechanical interlocking but also enhanced the optical and electronic properties of the material. Compared to its nonrotaxanated analogue TpV, the VCB COF exhibited a 3-fold increase in photoluminescence intensity and a stable photocurrent density of 1.3 μA cm^–2^ under 405 nm illumination. More significantly, the radical species within VCB demonstrated exceptional air stability, with ∼72% of the EPR signal retained after 7 days of exposure. These findings underscore the synergistic role of radical pimerization and macrocycle-mediated encapsulation in stabilizing reactive species within a crystalline porous framework. The successful incorporation of stable viologen radicals into a mechanically interlocked COF framework provides a promising strategy for the development of multifunctional materials with potential applications in energy storage, photodetection, photoresponsive devices, and air-tolerant electrochromic systems.

## Supplementary Material



## Notes

The Supporting Information is available free of charge. Materials and methods, synthesis, and characterization data of EPR, UV–vis, quantitative ^13^C NMR, PXRD, Raman, UV–vis–NIR DRS, photoluminescence, TGA, BET, EPR, and simulation supplementary details (S1–S20) (PDF)
