# Supplementary material for: Mechanically Interlocked Double-Walled Covalent Organic Framework with Cucurbit[8]Uril-Stabilized Viologen Radical Dimers
Source: J Am Chem Soc. 2026 Jul 23;148(30):31739–50. doi: 10.1021/jacs.6c01520 (PMC13449948; doi:10.1021/jacs.6c01520)
Supplement: Supplementary file 1 [file ja6c01520_si_001.pdf]

Supporting Information

Mechanically Interlocked Double-Walled Covalent Organic Framework with Cucurbit[8]uril-Stabilized Viologen Radical Dimers

Salma Abubakar,[1†] Bikash Garai,[1,2,3†] Nour Alkhatib,[1] Rasha G. AbdulHalim,[1,2] Thirumurugan Prakasam,[1] Sudhir Sharma,[4] Lydia Gkoura,[1] Ribal Jabbour,[1,5] Asif Equbal,[1,5] Felipe Gándara,[6] Jihan Dhainy,[7] Devin Thrush,[8] Claudia E. Avalos,[8] Gobinda Das,[1] Sharath Kandambeth,[9] Na'il Saleh,[10] Ramesh Jagannathan,[4] Mark A. Olson,[11] Mohamad El-Roz,[7] Serdal Kirmizialtin,[1*] Mohamed Eddaoudi,[9*] and Ali Trabolsi [1,2*]

[1] Chemistry Program, New York University Abu Dhabi, Saadiyat Island, Abu Dhabi 129188 PO Box, UAE

[2] Water Research Centre, New York University Abu Dhabi, Saadiyat Island, Abu Dhabi 129188 PO Box, UAE

[3] Department of Chemistry and Chemical Biology, Indian Institute of Technology (Indian School of Mines) Dhanbad, Dhanbad 826004, Jharkhand, India

[4] Engineering Division, New York University Abu Dhabi, Saadiyat Island, Abu Dhabi 129188 PO Box, UAE

[5] Center for Quantum and Topological Systems, New York University Abu Dhabi, Saadiyat Island, Abu Dhabi 129188 PO Box, UAE

[6] Materials Science Institute of Madrid, CSIC, Sor Juana Ine´s de la Cruz 3, Madrid 28049, Spain

[7] Catalysis and Spectrochemistry Laboratory, CNRS, ENSICAEN, Caen University, Caen cedex 14050 PO Box, France

[8] Department of Chemistry, New York University, New York 10003, New York, United States

[9] Physical Science and Engineering Division, King Abdullah University of Science and Technology, Thuwal 23955-6900, Saudi Arabia

[10] Department of Chemistry, College of Science, United Arab Emirates University, Al Ain 15551 PO Box, UAE

[11] Department of Physical & Environmental Sciences, Texas A&M University Corpus Christi, Corpus Christi 78412, Texas, United States

*Correspondence Email: mohamed.eddaoudi@kaust.edu.sa, serdal@nyu.edu, ali.trabolsi@nyu.edu

†S. A., and B. G. contributed equally to this work.

Table of contents

1. Materials

2. Instruments and Methods

3. Synthesis of VCB COF

4. Characterization of the [3]Pseudorotaxane CB[8]⊃$(V^{\bullet+})_2$

Figure S1: EPR spectra of CB[8]⊃$(V^{\bullet+})_2$ and its precursors

Figure S2: UV-Vis spectra of the CB[8]⊃$(V^{\bullet+})_2$ and its precursors

Figure S3: Representative illustration for synthesis of VCB through interfacial approach

5. Characterization of the VCB COF

Figure S4: Fourier-transform infrared spectroscopy (FT-IR) spectra

Figure S5: Solid-state NMR of VCB and its precursors

Figure S6: Solid-state ^{15}N CP-MAS spectrum analysis

Figure S7: Morphology of VCB synthesized through interfacial approach

Figure S8: Diffuse Reflectance Spectroscopy (DRS) of VCB and TpV

Figure S9: Thermogravimetric analysis (TGA)

Figure S10: Simulation setup structure of VCB

Figure S11: DFT Analysis of Structural Candidates

Figure S12: QMD Analysis of Framework Thermal Stability

Figure S13: Porosity and surface area analysis

Figure S14: Preliminary AFM scans and marking for mechanical properties

Table S1: Mechanical properties comparison between VCB, TpV, and TpVCB[7]

Figure S15: EPR spectrum of VCB with SDT

Figure S16: EPR spectrum of VCB and reduced TpV through exposure to air

Figure S17: EPR spectrum of reduced TpV, after CB[8] adsorption and when exposed to air

Figure S18: FT-IR spectrum of reduced TpV and CB[8] adsorption

Figure S19: Photocurrent density versus time for VCB and TpV

Figure S20: Photoluminescence of VCB and TpV

1. Materials

The starting materials and solvents used for the synthesis of Tp, VNO_2·2Cl, and VNH_2·2Cl, were purchased from Sigma-Aldrich, Fisher Scientific, and Laboratory Scientific Supplies (LSS) and were utilized without further purification. The synthesis of 1,1'-bis(2,4-dinitrophenyl)-[4,4'-bipyridine]-1,1'-diium dichloride (VNO_2·2Cl), which is a precursor to the1,1'-bis(4-aminophenyl)-[4,4'-bipyridine]-1,1'-diium dichloride (V^{2+}) linker used for VCB COF synthesis, was conducted following a previously reported procedure, with the product being purified by washing with acetonitrile (50 mL, one time) and diethyl ether (40 mL, 3 times).[1] Moreover, 2,4,6-triformylphloroglucinol (Tp) and 1,1'-bis(4-aminophenyl)-[4,4'-bipyridine]-1,1'-diium dichloride (V^{2+}), which were used to synthesize VCB, were also prepared following previously reported procedures.[2, 3]

2. Instruments and methods

2.1 Solution state proton Nuclear Magnetic Resonance (^{1}H NMR) spectra were recorded on a Bruker 500 MHz Avance spectrometer at room temperature operating at a frequency of 500 MHz. The chemical shifts (ppm) are reported relative to the signals corresponding to the residual non-deuterated solvents ($CDCl_3$: δ = 7.24 ppm, CD_3OD: δ = 3.31 ppm, and D_2O: δ = 4.97 ppm).

2.2 Fourier Transform Infrared (FT-IR) spectroscopic characterizations were carried out on Agilent FT-IR 670/630 spectrometer.

2.3 Thermogravimetric Analysis (TGA) was performed on TA SDT Q600 analyzer over the temperature range of 30-900 °C under nitrogen atmosphere with a heating rate of 10 °C min^{-1}.

2.4 Solid-state NMR experiments were performed on a Bruker Avance-HD 600 MHz Wide Bore WB spectrometer operating at a magnetic field of 14.1 T (^{1}H Larmor frequency, $\omega_{1H}/(2\pi) = 600\,\mathrm{MHz}$, ^{15}N Larmor frequency $\omega_{15N}/(2\pi) = 60.8\,\mathrm{MHz}$, ^{13}C Larmor frequency $\omega_{13C}/(2\pi) = 150.9\,\mathrm{MHz}$). A 4.0 mm Magic Angle Spinning (MAS) probe was used for all the room temperature measurements. Powdered samples were packed into 4.0 mm zirconia rotors. MAS frequency of 14 kHz was used for high resolution spectroscopy. One-dimensional (1D) ^{13}C cross-polarization (CP) spectra were recorded on all materials. ^{1}H-^{13}C CP-MAS experiments were performed using a standard linearly ramped cross-polarization pulse on ^{1}H and CP mixing time was set to 2 ms and RF power was 45 W. Heteronuclear decoupling was used to during signal acquisition to improve spectral resolution. The ^{13}C chemical shifts were externally referenced to the adamantane CH_2 signal at 38.48 ppm. All CP-MAS experiments performed using a recycle delay of 5 s and the ^{13}C direct excitation MAS experiments were carried out using a recycle delay of 20 s. All the NMR data were processed using TopSpin software.

2.5 Dynamic Nuclear Polarization (DNP): ^{1}H-^{13}C DNP/CP-MAS and ^{13}C direct excitation experiments were collected at New York University Abu Dhabi on a 600 MHz (14.1 T) Bruker Avance-HD solid-state NMR spectrometer. The spectrometer is equipped with a low temperature magic angle spinning (LTMAS) 3.2 mm triple resonance probe tuned to ^{1}H and ^{13}C resonances. The probe is coupled with a Bruker gyrotron 7.2 T producing 395 GHz continuous wave (CW) microwaves (µw). ^{1}H-^{15}N DNP/CP-MAS experiments were collected at the New York Structural Biology Center (NYSBC) on a similar Bruker Avance III 600 MHz spectrometer operating at a magnetic field of 14.1 T using a 3.2 mm LTMAS probe. All samples used in this work were first impregnated with 10 mM AMUPOL or ASYMPol-Pok in DNP juice (60: 30: 10 d_8 glycerol: D_2O: H_2O by volume), with ^{1}H-^{13}C DNP/CP-MAS and ^{13}C direct excitation samples left for 48 h to allow

the matrix molecules to disperse uniformly. The samples were packed into 3.2 mm sapphire rotors as they are transparent to microwave. DNP experiments were carried out at 10 kHz spinning and at 100 K temperature. A recycle delay of 3 s and CP contact time of 2 ms was used for ^{1}H-^{13}C measurements. These values were 5.6 s and 3 ms, respectively, for ^{1}H-^{15}N measurements. Heteronuclear decoupling was used in all the measurements. For all ^{1}H-^{15}N CP experiments ^{15}N chemical shifts were referenced to ^{15}N enriched u-glycine (33.4 ppm).[4] For the ^{13}C direct excitation DNP MAS experiments were carried out at 10 kHz using a recycle delay of 20 s. 1500 scans were recorded for ^{13}C direct excitation and 256 scans for ^{1}H-^{13}C CP-MAS experiments.

2.6 Electron Paramagnetic Resonance (EPR) Spectroscopy analyses were conducted using Bruker bench-top EMXnano EPR system under the frequency of 9.6 GHz at room temperature, with a centerfield at 3400 G, sweep width of 400 G, sweep time of 20 s, and a microwave power attenuation of 25 dB.

2.7 UV-Vis Absorbance spectra were collected using UV-Vis-NIR UV-3600i Shimadzu spectrophotometer equipped with photomultiplier tube (PMT) and an InGaAs detector and processed using LanSolutions UV-Vis software.

2.8 UV-Vis-NIR Diffuse-Reflectance Spectroscopy (DRS) spectra of the material were obtained by using the Kubelka-Munk conversion [$\alpha/S= (1 - R_{\infty})2/2R_{\infty}$ (where, α is absorption coefficient and S is scattering coefficient and $R_{\infty} = R_{sample}/R_{standard}$)] at room temperature for the solid samples on a SHIMADZU UV-3600 (Kyoto, Japan) equipped with an integrating sphere as the sample holder. To avoid the reflection from sample surface the incident light was pointed to the sample at an angle of 0°. Thus, scattering coefficient (S) = 1. Barium sulphate ($BaSO_4$) was used as the standard.

2.9 Scanning Electron Microscopy (SEM) images were obtained from ThermoFischer Quanta3D Scanning Electron Microscope. Samples were prepared by dropping the dispersed COF in ethanol onto a silicon surface and allowing the sample to dry prior to imaging.

2.10 High Resolution Transmission Electron Microscopy (HR-TEM) images and diffraction patterns were obtained using a ThermoFischer TAlos F200X HR-TEM. The samples were prepared by dropping dispersed COF in ethanol onto carbon grids coated on 400 mesh copper and allowing the samples to dry prior to imaging.

2.11 AFM imaging and Nano-mechanical testing of VCB nanosheets: Both Force vs Distance (F/D) and load-displacement (P–H) curves were also measured using an atomic force microscope, Park NX10, Park Systems Korea in true non-contact mode for topological investigations. SmartScanTM software developed by Park Systems, Korea was used to collect the AFM scans. Ultra-sensitive AFM probes (Super Sharp Standard NCH cantilevers, High Density Carbon tip, Manufacturer: NanoWorld AG, Switzerland) with a typical tip height of 10-15 µm and a 2 nm tip radius were used for collecting the AFM scans under ambient conditions. These cantilevers were 125 µm in length, 30 µm in width, 4 µm in thickness, with 42 $N \cdot m^{-1}$ force constant value and ~320 kHz resonant frequency, respectively. AFM scans were collected with a resolution of 1024 lines/image × 1024 points/line. For the F/D measurements, the vertical force exerted by the AFM tip to the sample surface during contact-AFM imaging. The deflection of the cantilever versus the extension of the piezoelectric scanner is directly correlated to the tip-sample interaction forces.

2.12 Brunauer-Emmett-Teller (BET) method was used to analyze the surface properties of VCB. N_2 adsorption/desorption isotherms were obtained via a manometric Micromeritics 3Flex gas sorption analyzer at 77 K at pressures up to 1 bar. The sample was fully degassed and activated prior to measurement using a degas station by heating at 100 °C for 20 h. The pore distribution was calculated using the Nonlocal Density Functional Theory (NLDFT).

2.13 Time Resolved Photoluminescence (TR-PL) spectra were collected after excitation at 355 nm with a Nd:YAG pulsed laser (5 ns/pulse). All the samples were prepared with an optical density of around 0.2 at the excitation wavelength.

2.14 Photoelectrochemical Experiments were performed by using a Autolab potentiostat PGSTAT302N in a two-compartment cell and three-electrode configuration. The cathodic compartment was physically separated from the anodic side by a proton exchange membrane (Nafion 117; thickness 0.01778 cm. The fluorine-doped tin oxide-covered glass (FTO) was utilized as a conductive substrate for the working electrode (geometric area 0.71 cm^2), Pt wire was employed as the counter electrode, and a mercury sulfate electrode (Hg/Hg_2SO_4/sat. K_2SO_4) as the reference electrode. The aqueous electrolyte solution consisted of 0.1 mol·dm^{-3} $Na_2S_2O_4$. The electrochemical experiments were performed at different scan rates of 50 mV·s^{-1}. Catalyst solutions were prepared by suspending 10 mg of photocatalyst in a mixture of 200 µL Nafion solution (0.5 wt%) and 20 µL of ethanol, followed by sonification for 30 min. Thereafter, 20 µL of the sample solution was pipetted onto the FTO surface (geometric area 0.71 cm^2) followed by drying at 60 °C for 60 min. For photoelectrochemical experiments, a light-emitting diode (LED) emitting at $\lambda = 405$ nm was used as a light source. The photocurrent vs. time curves were measured at an imposed potential of 1 V vs. SCE under chopped light.

2.15 Molecular Dynamic Simulation methodology: All-atom molecular dynamics simulations were executed employing the LAMMPS software.[5] Based on CIF files, the VCB COF structure and CB[8] macrocycles were positioned. The VCB COF structure was expanded into a multilayer configuration with a unit cell dimension of 90 x 90 x 36 Å^3, and varying numbers of CB[8] macrocycles were positioned on the nitrogen sites of the COF. Four distinct system configurations were established, encompassing two, six, eight, and twelve macrocycles. For each macrocycle, 2 Cl^- ions were added to maintain charge neutrality. The Dreiding-x6 force field,[6] which has demonstrated good agreement with experimental data in our previous studies,[7-9] was employed to model the interactions between the COF and macrocycle. For each system, a geometry optimization was performed using the steepest descent method for 5000 steps, allowing the system to attain its minimum energy state. Subsequently, molecular dynamics simulations were carried out in an NPT ensemble, where the temperature was set to 298.15 K and the pressure was maintained at 1 atm. Periodic boundary conditions were applied in all directions for volume and shape fluctuations during the MD simulations. Pairwise interactions were truncated at a cutoff of 12 Å, and long-range Coulombic interactions were computed using Ewald summation with an accuracy of 1×10^{-5} kJ·mol^{-1}.[10] A time step of 1 fs was utilized, and the simulations were set to run for 100 ns, with monitoring of the convergence of the structural ensemble. Additionally, to compute the dipole moment of CB[8] the structural arrangement of the CB[8] molecule was optimized using NWChem 6.6.[11] This optimization process utilized the Hartree-Fock theory in conjunction with 6-31G* basis set. All parameters and criteria pertaining to the minimization and convergence were maintained at their default values during the computations. The dipole moment of the macrocycle was computed from the energy minimized structure.

Energy calculations for intermediate conformations: To assess the formation energies of intermediate states, we constructed the intermediate states as described in Figure S11. Each structure is then geometry optimized using density functional theory calculations with the CASTEP code[12] employing the Perdew–Burke–Ernzerhof (PBE) exchange–correlation functional.[13] A plane-wave basis set with a kinetic energy cutoff of 489.8 eV was used, together with a real-space grid defined by a grid scale of 1.5. Core–valence interactions were treated using standard pseudopotentials as implemented in CASTEP. Geometry optimizations were conducted using the Broyden–Fletcher–Goldfarb–Shanno (BFGS) algorithm with a speed-optimized convergence strategy to enable efficient pre-relaxation of large frameworks.[14] Electronic minimization employed a density mixing scheme with Pulay mixing, and a convergence threshold of 0.00001 eV per atom was applied for the self-consistent field (SCF) cycle. Structural convergence criteria were set to 0.0001 eV for total energy, 0.10 eV $Å^{-1}$ for forces, and 0.005 Å for atomic displacements. A maximum of 80 SCF cycles and 80 geometry optimization steps were allowed.

This protocol was applied consistently across all systems, including the isolated macrocycle, the single-layer COF, the double-layer COF and the macrocycle embedded within single-layer and double-layer COF environments, to extract the corresponding formation energies.

Thermal stability analysis of VCB: To assess the thermal stability of the CB[8]–viologen complex and the possibility of bond cleavage, molecular dynamics simulations were performed on a representative local VCB structure using the semi-empirical GFN2-xTB tight-binding method,[15] as implemented in the *xtb* program,[16] following previous studies.[17-20]

All simulations were carried out using the GFN2-xTB Hamiltonian in the canonical (NVT) ensemble at elevated temperatures. Simulations were performed at 650, 1000, 1500, and 2000 K to probe the temperature dependence of structural stability and to identify the onset of bond cleavage under progressively more extreme conditions. The simulations at 650 K correspond to the experimentally relevant decomposition regime, while higher temperatures were employed to accelerate rare bond-breaking events and to identify dominant degradation pathways. Each simulation was performed for 50 ps with an integration time step of 2.0 fs. No bond constraints were applied. Temperature was controlled using a Nosé–Hoover thermostat, and trajectory configurations were recorded every 50 fs for analysis.

2.16 Radical air-stability experiment: 5 mg of VCB and reduced TpV were placed in 4 mm thin-wall quartz EPR sample tubes, and their EPR signal was monitored daily via Bruker bench-top EMXnano. The reduced TpV was prepared by exposing the material to a 0.5 mL aqueous solution containing sodium dithionite (SDT, 2 molar eq of V^{2+} units in TpV) inside the glove box.

For the control study, TpV was suspended in deoxygenated water and reduced by adding SDT. The reduced COF was then added to aqueous CB[8] solution and the solid was collected for FT-IR and EPR analysis.

3. Synthesis of VCB COF

The synthesis of VCB COF was conducted inside a glove box to maintain inert conditions. The [3]pseudorotaxane CB[8]$\supset$($V^{\bullet+}$)$_2$ were prepared by first dissolving V^{2+} (5.1 mg, 0.012 mmol) in 5 mL of degassed distilled water. Then, SDT was added to the solution (10.79 mg, 0.061 mmol) before stirring the solution for few minutes to ensure the reduction of all the organic monomers. Afterwards,

CB[8] was added to the solution (8.2 mg, 0.006 mmol) and the mixture was stirred for 1 h to allow complexation of the dimers, which was indicated by the color change to dark brown-black (solution A). Then, Tp (3.9 mg, 0.018 mmol) was dissolved separately in 5 mL of dichloromethane (solution B). In a 20 mL glass vial, solution B was first layered, followed by the layering of degassed H_2O dropwise on top of solution B. Then, *p*-Toluenesulfonic acid (PTSA) was added to solution A (9.6 mg, 0.055 mmol). This aqueous solution was then layered on top of the water layer dropwise and slowly over a duration of 15 minutes. The assembled system was left undisturbed inside the glove box for 72 h at room temperature, after which a dark brown film formed at the liquid interface. The film was later extensively washed with water and then washed with acetone (17% yield). Finally, the product was dried in vacuo before further characterization.

For the solvothermal synthesis, 5.1 mg of V^{2+} and 10.79 mg SDT were taken in a tube and added with 1.5 ml degassed DMAc and 0.5 ml degassed water. The solution was sonicated to dissolve the reactants, and then 8.2 mg CB[8] was added to it. The resulting solution was sonicated for 30 min to obtain a homogenous mixture and then added with 3.9 mg Tp, and 9.6 mg PTSA. The tube was then sealed and subjected to 3 freeze-pump-thaw cycles. The tube was then heated at 120 °C for 3 days. The obtained solid was then collected and purified by washing with water and diluted HCl to obtain a final yield of 55%.

4. Characterization of the [3]Pseudorotaxane CB[8]$\supset$($V^{\bullet+}$)$_2$

EPR spectra of the CB[8]$\supset$($V^{\bullet+}$)$_2$ and its precursors

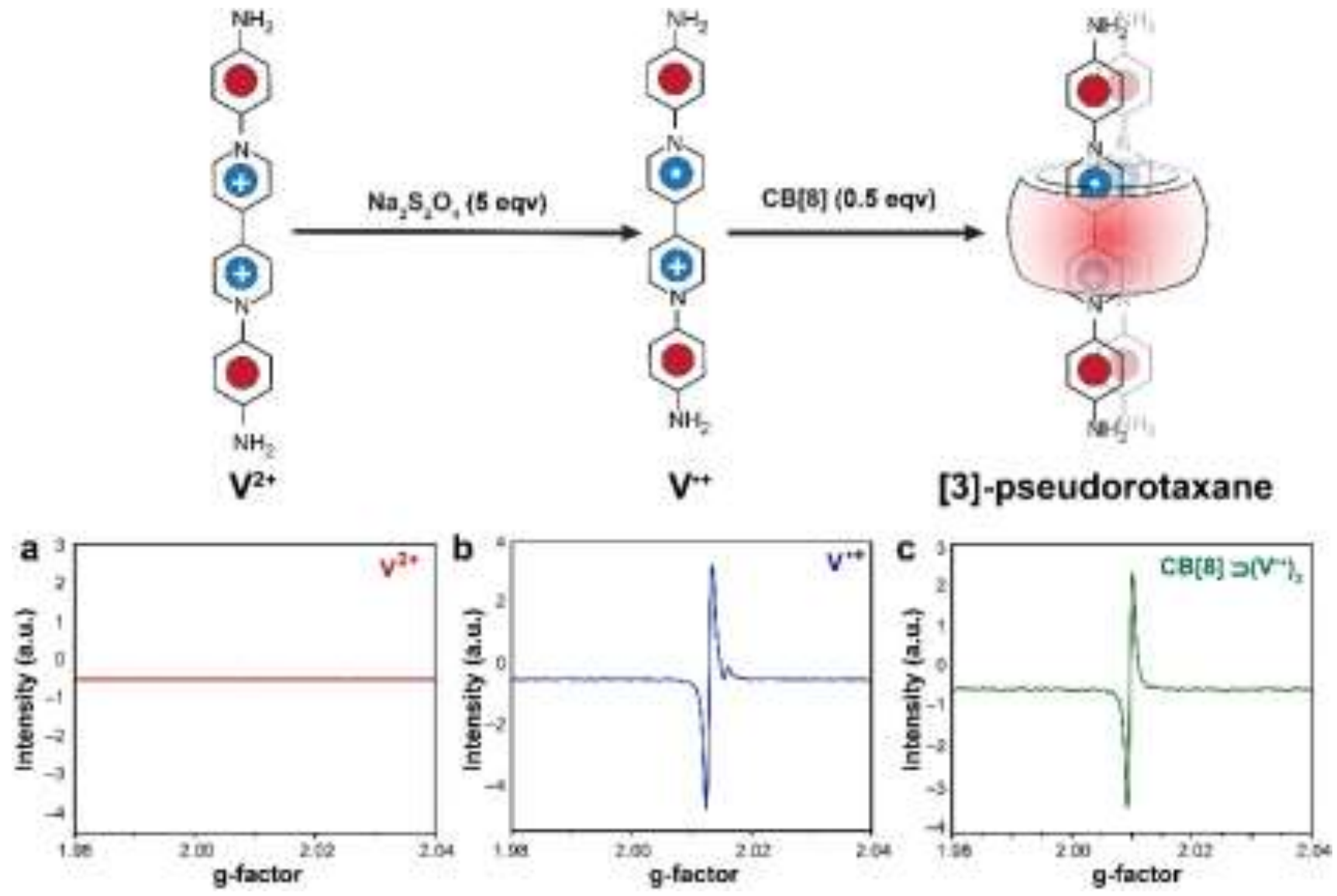

Figure S1: Electron paramagnetic resonance (EPR) spectra of (a) the viologen dication (V^{2+}), (b) the reduced viologen radical cation ($V^{\cdot+}$), and (c) CB[8]$\supset$($V^{\bullet+}$)$_2$ formed by the encapsulation of viologen radical cation dimers within CB[8]. No EPR signal is observed for V^{2+} (a), while a strong radical signal appears for $V^{\cdot+}$ (b), confirming successful reduction. In (c), CB[8]$\supset$($V^{\bullet+}$)$_2$ shows a slightly modified EPR signal, indicating the formation of the host–guest complex and stabilization of the radical species within the macrocyclic cavity.

UV-Vis spectra of CB[8]$\supset$($V^{\bullet+}$)$_2$ and its precursors

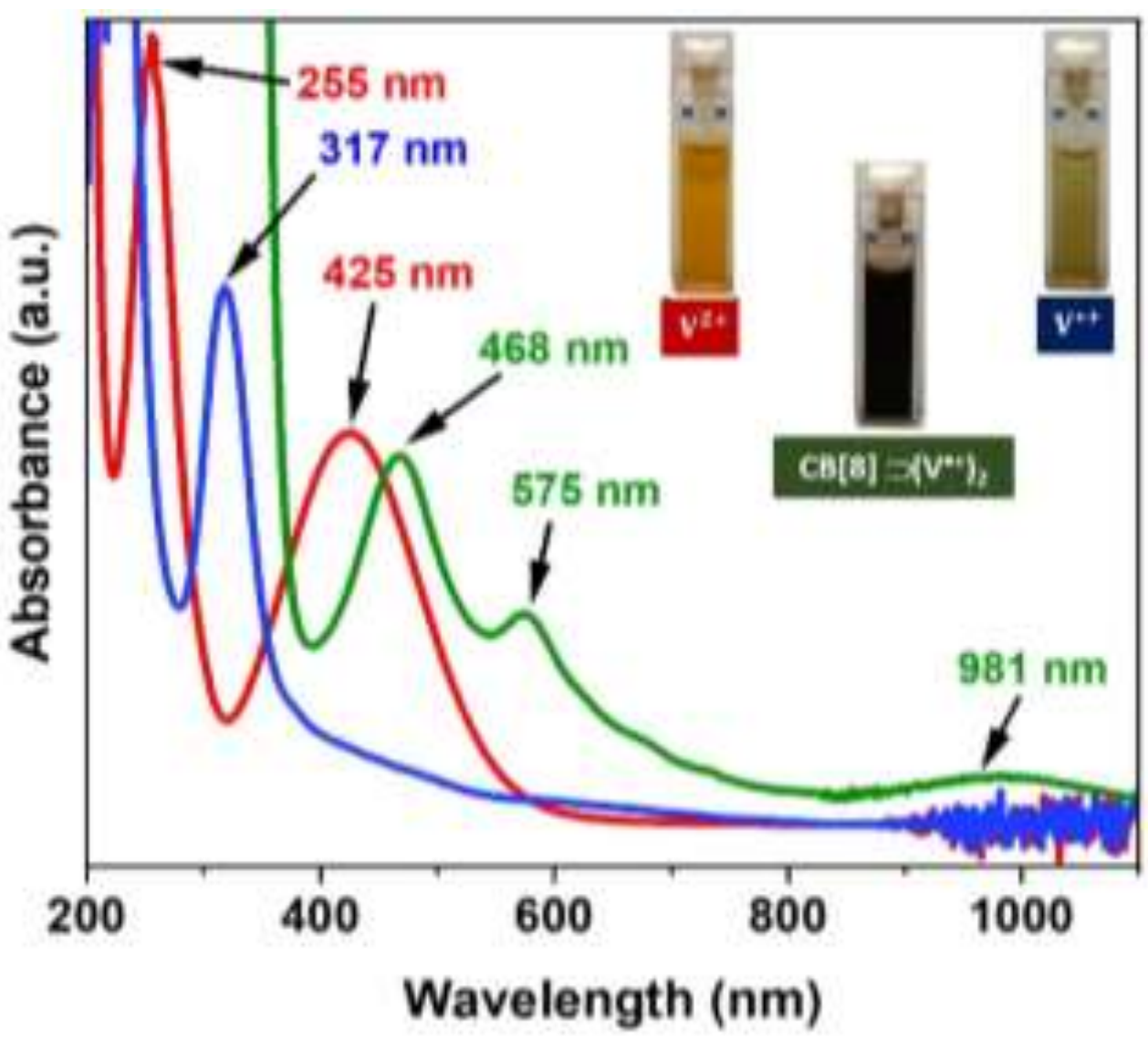

Figure S2: Overlaid UV-Vis absorption spectra of aqueous solutions containing 0.2 mM viologen dication (V^{2+}, red), viologen radical cation ($V^{\cdot+}$, blue), and the [3]pseudorotaxane complex (green) formed by mixing $V^{\cdot+}$ with 0.5 molar equivalents of CB[8]. Spectra were recorded at room temperature using a 5 mm path length cuvette.

Interfacial synthetic scheme

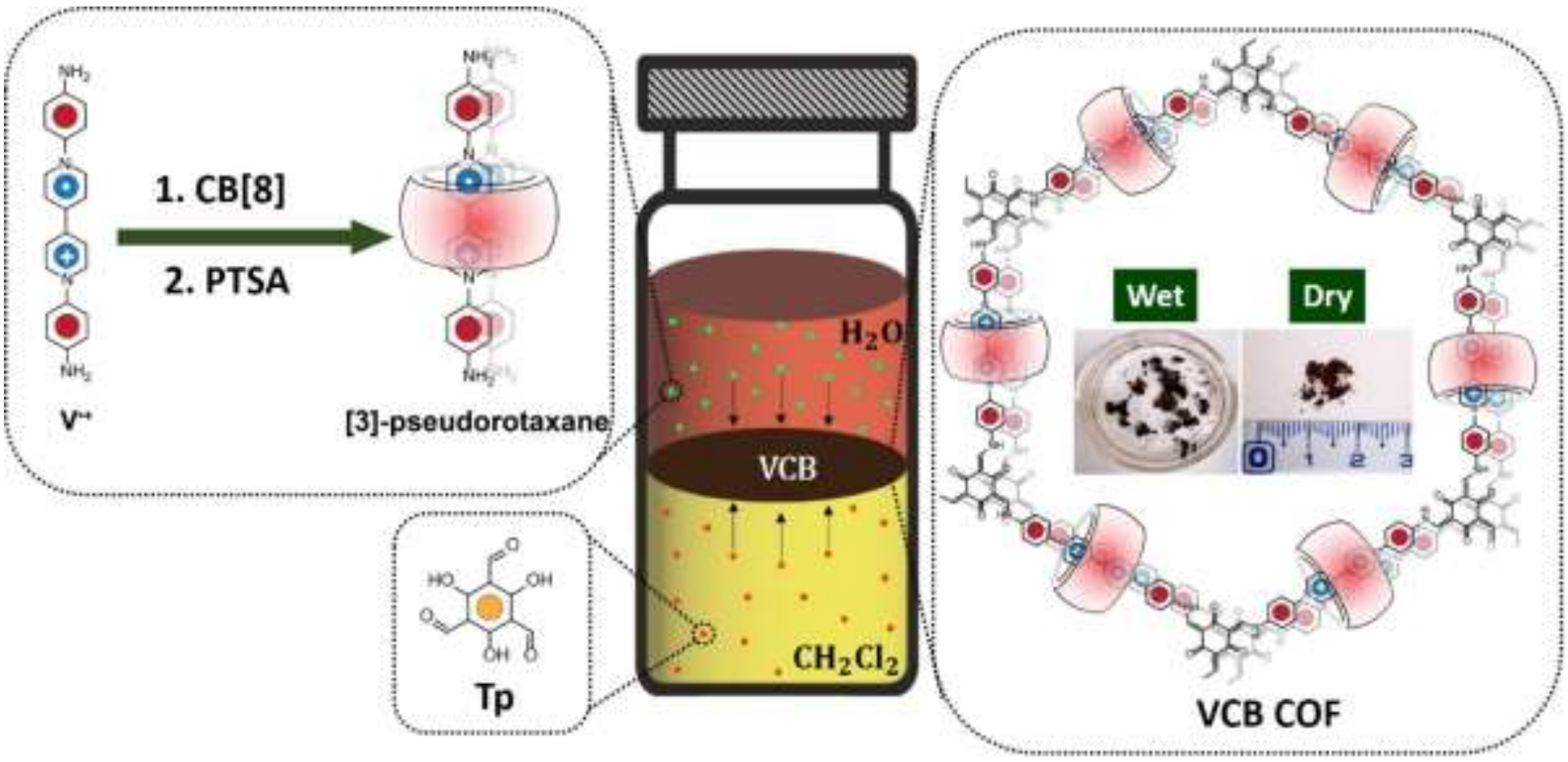

Figure S3: Representative illustration for synthesis of VCB through interfacial approach.

5. Characterization of the VCB COF

Fourier-transform infrared spectroscopy (FT-IR) spectra

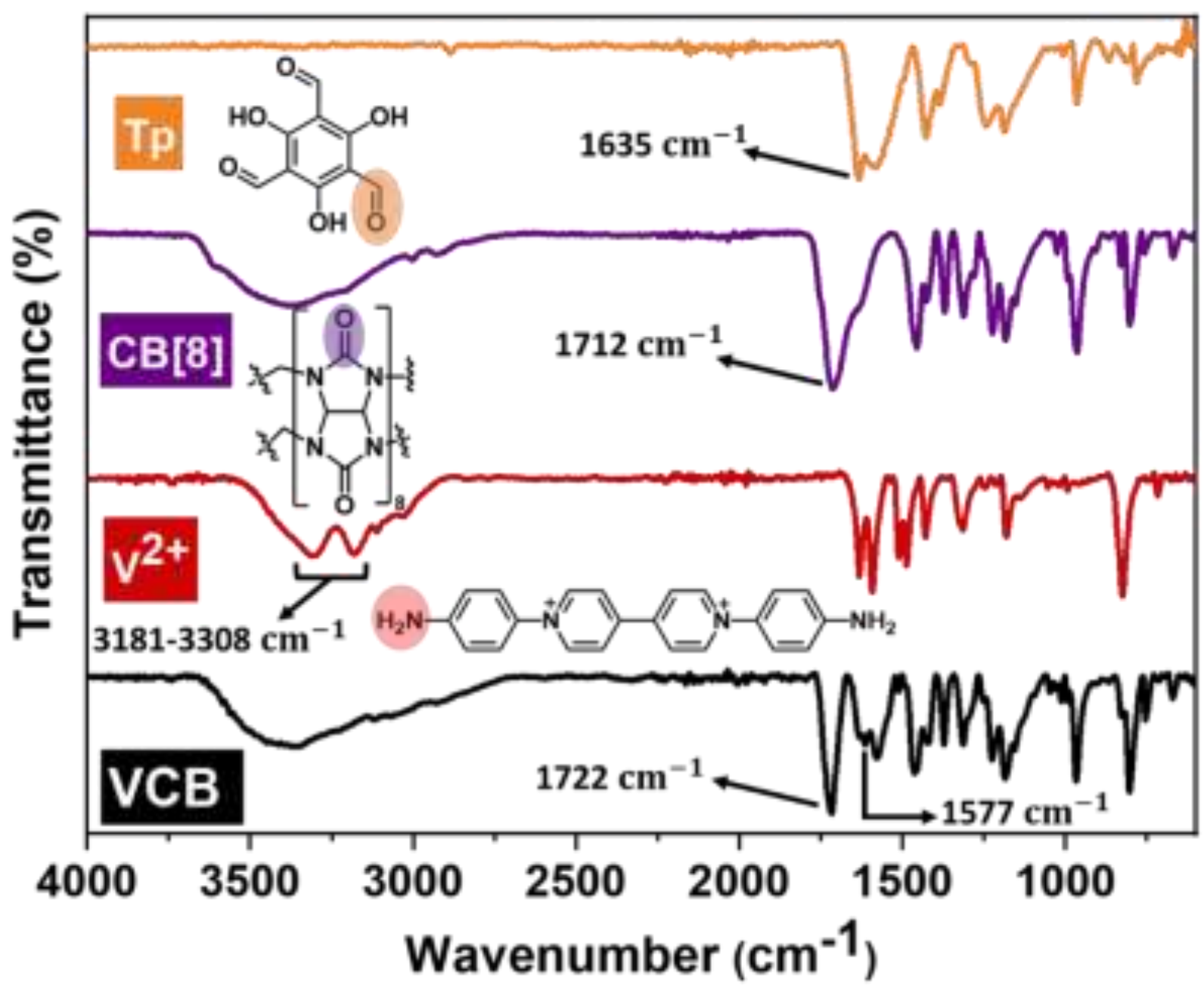

Figure S4: Stacked FT-IR spectra of VCB COF (black) and its individual components: Tp (orange), CB[8] (purple), and viologen dication (V^{2+}, red).

Solid-state ^{13}C NMR of VCB

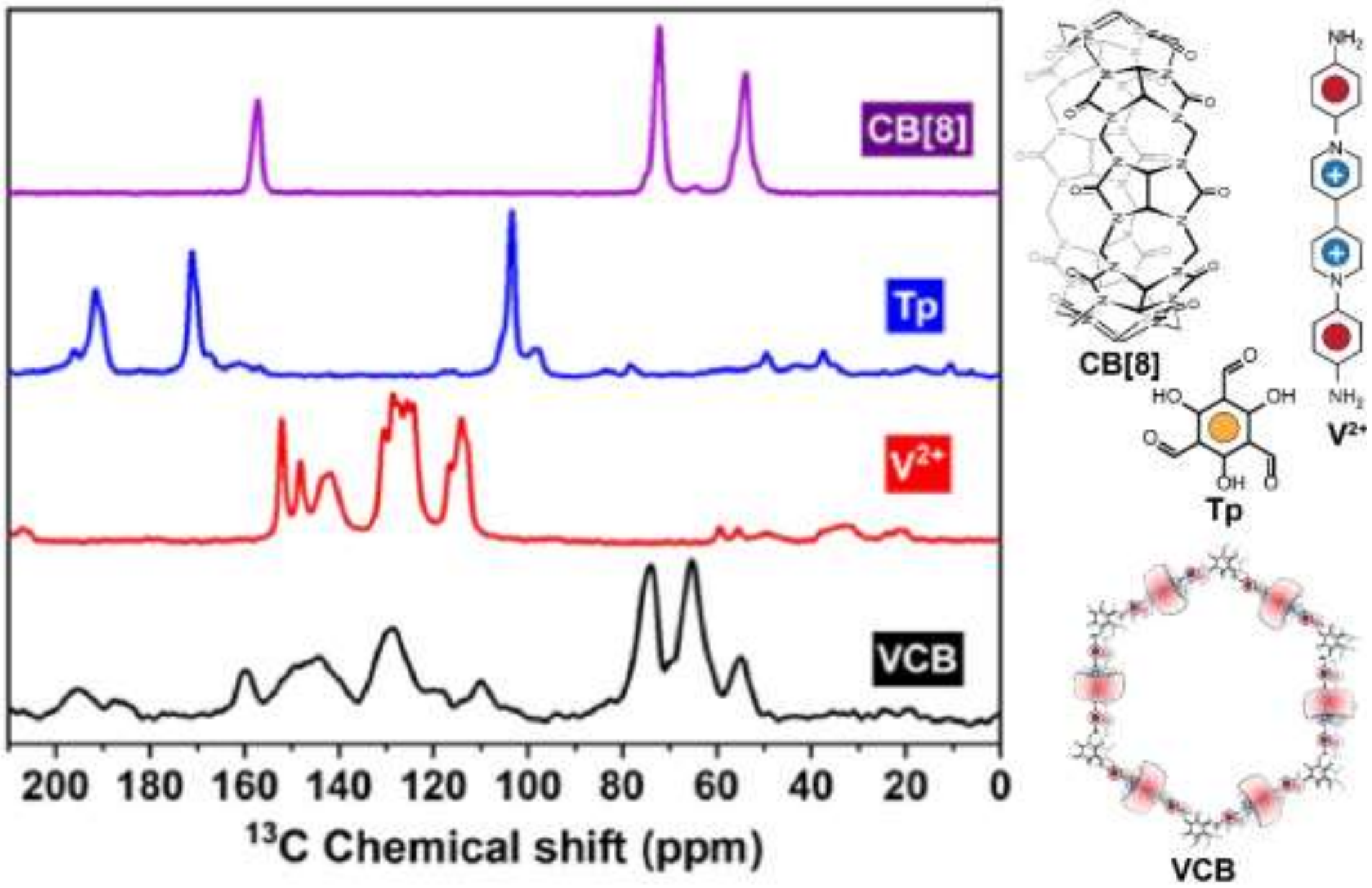

Figure S5: Solid-state ^{13}C NMR of VCB and its precursors. VCB displays broader CB[8] ^{13}C signals compared to free CB[8], consistent with host–guest interactions.

Solid-state ^{15}N DNP/CP-MAS spectrum of VCB

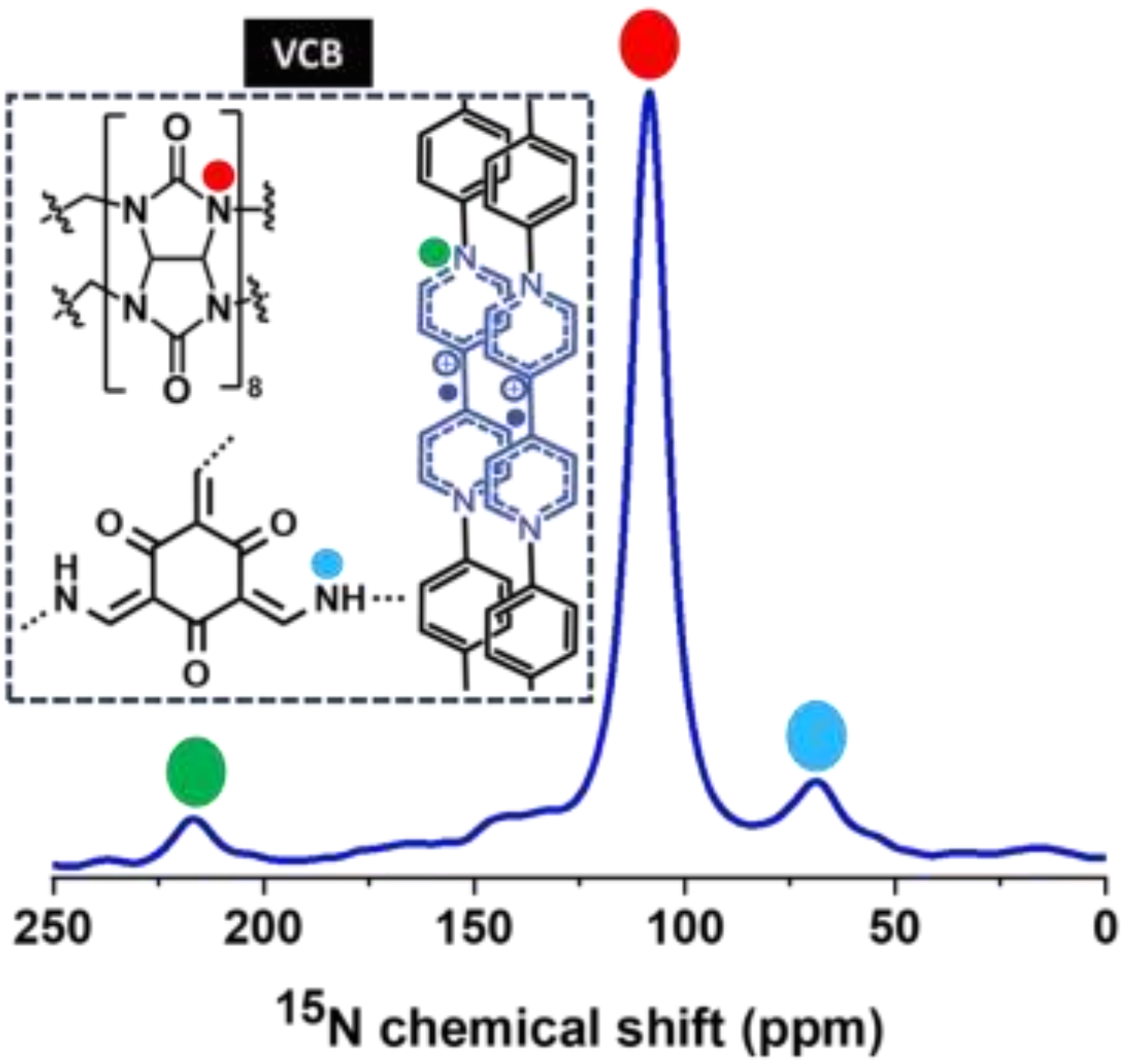

Figure S6: Solid-state ^{15}N DNP/CP-MAS spectrum of VCB showing signals for secondary amine (blue), tertiary amine (red), and iminium nitrogen on VCB (green).

SEM Morphology

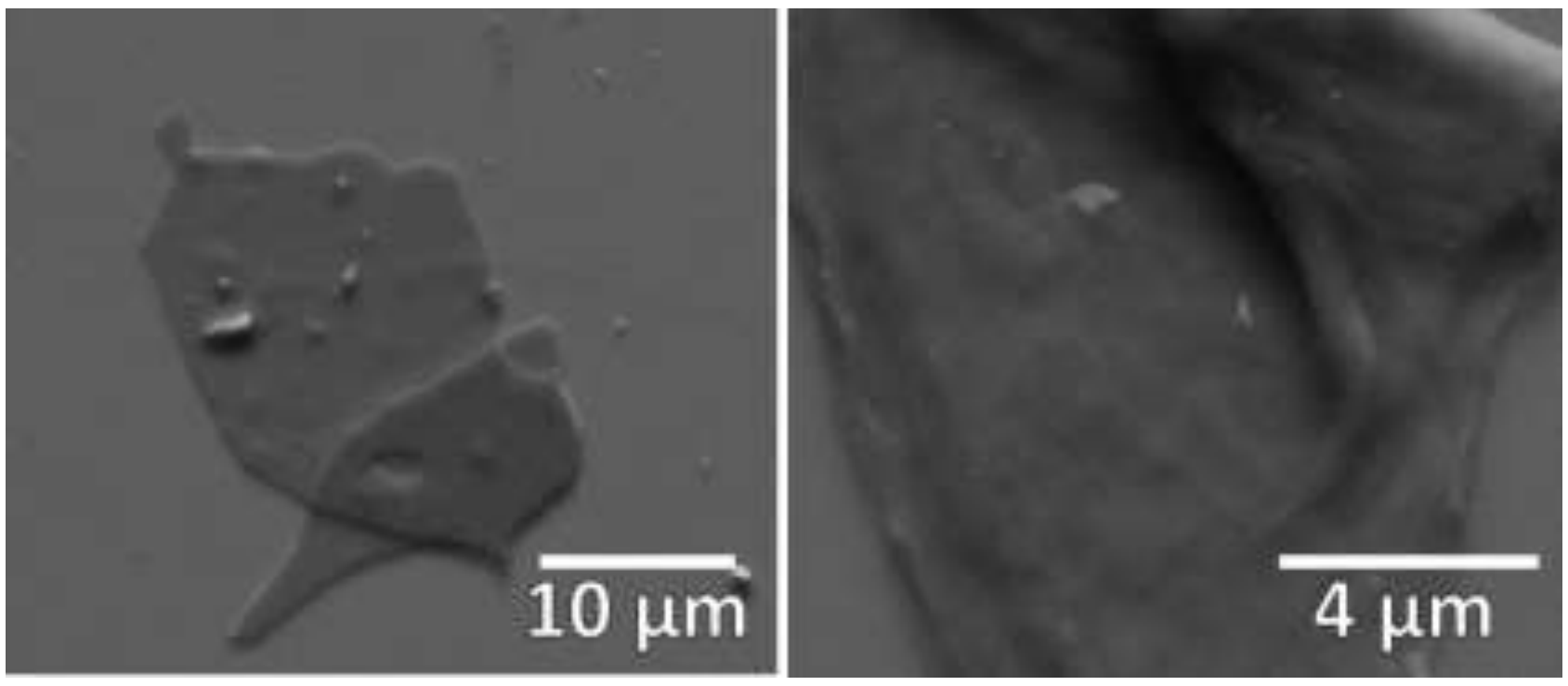

Figure S7: Morphology of VCB synthesized through interfacial approach, showing its film morphology.

Diffuse Reflectance Spectroscopy (DRS) of VCB and TpV

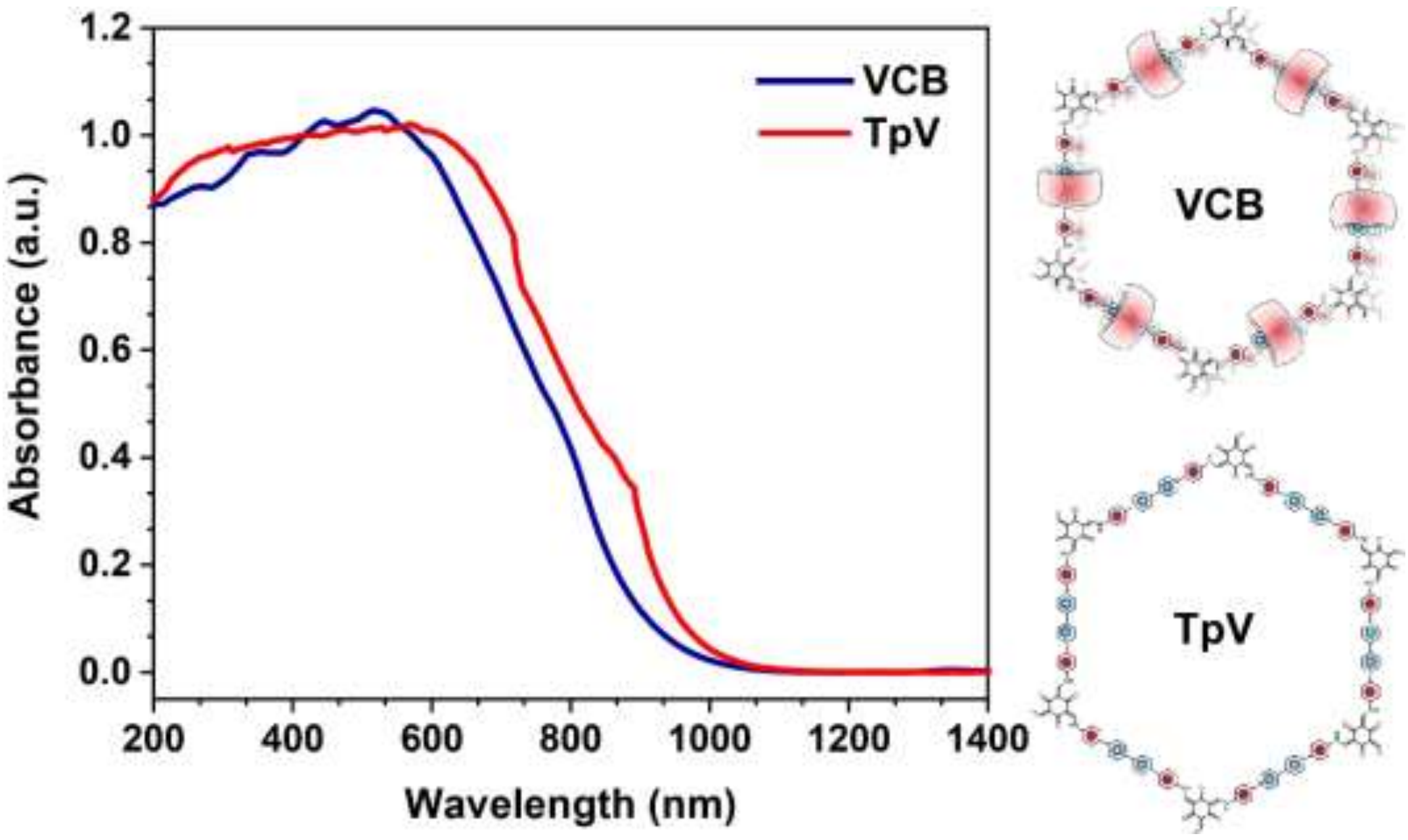

Figure S8: UV-Vis diffuse reflectance spectra (DRS) for VCB and TpV utilizing the Kubelka-Munk function.

Thermogravimetric Analysis (TGA)

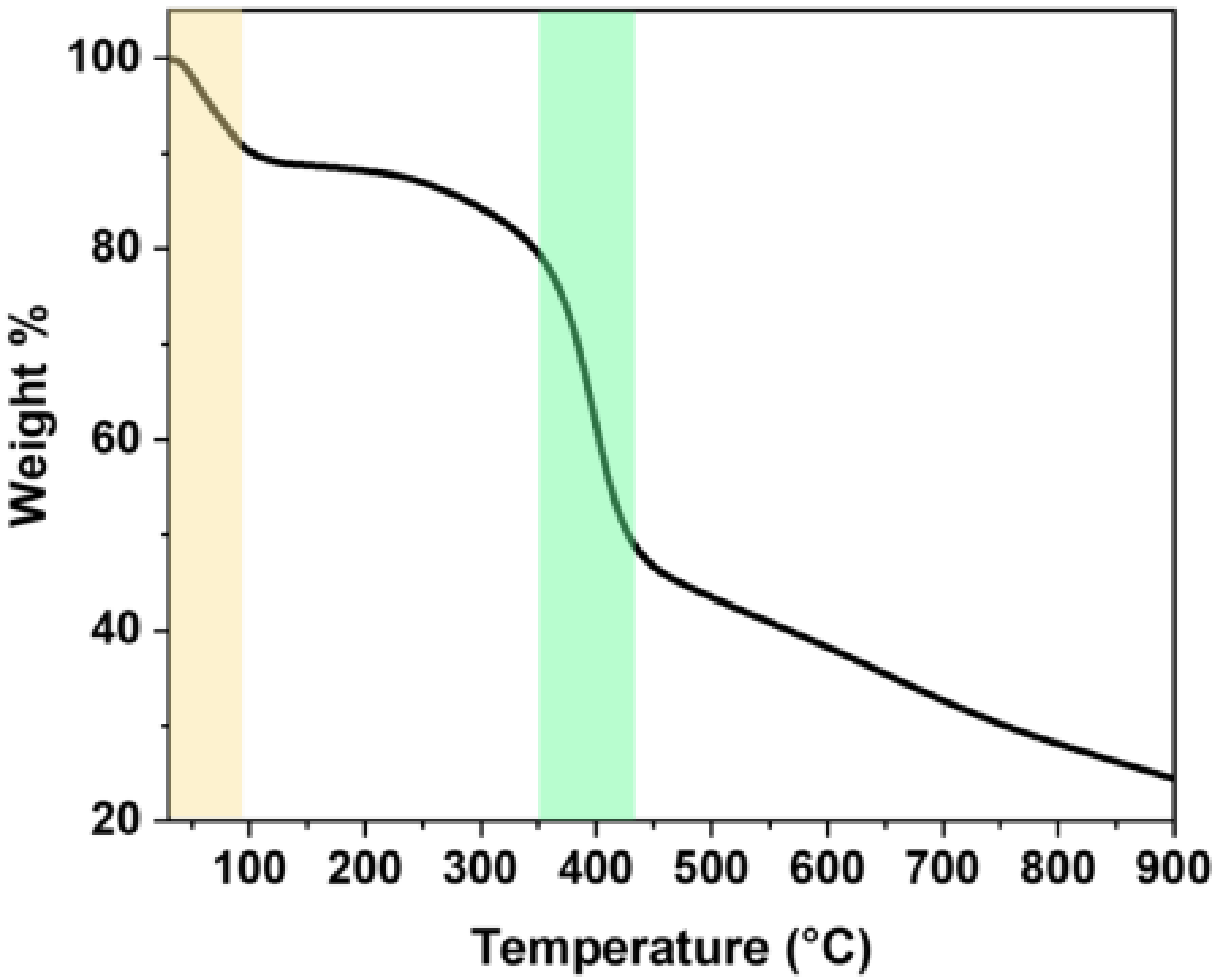

Figure S9: TGA plot of VCB recorded under N_2 atmosphere.

Simulation setup structure of VCB

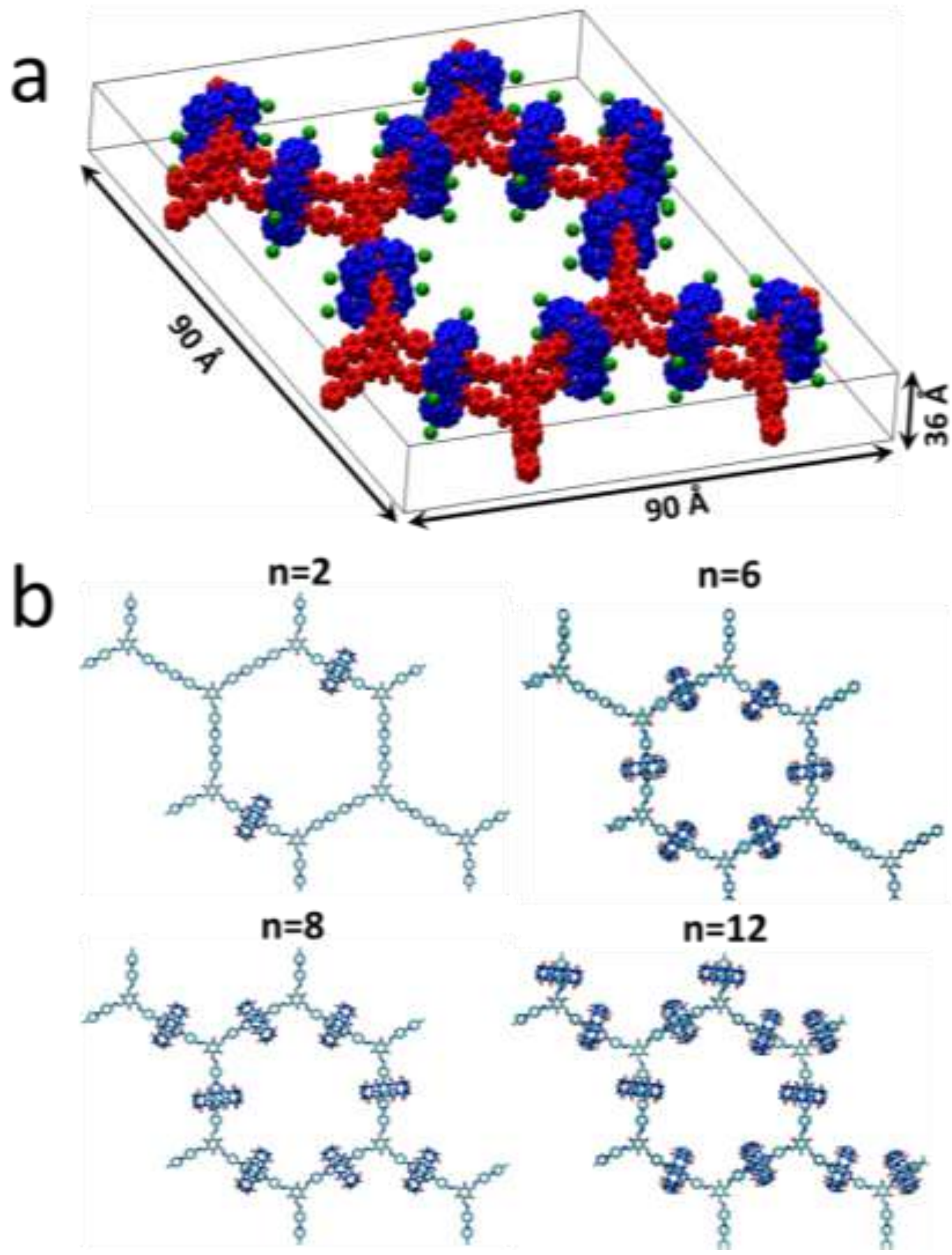

Figure S10: a) VCB structure setup used for the molecular dynamic studies, showing chloride ions (COF in red, macrocycles in blue, and chloride ions in green). b) The initial states the investigated COF systems with varying numbers of CB[8] macrocycles ranging from n=2, 6, 8, 12.

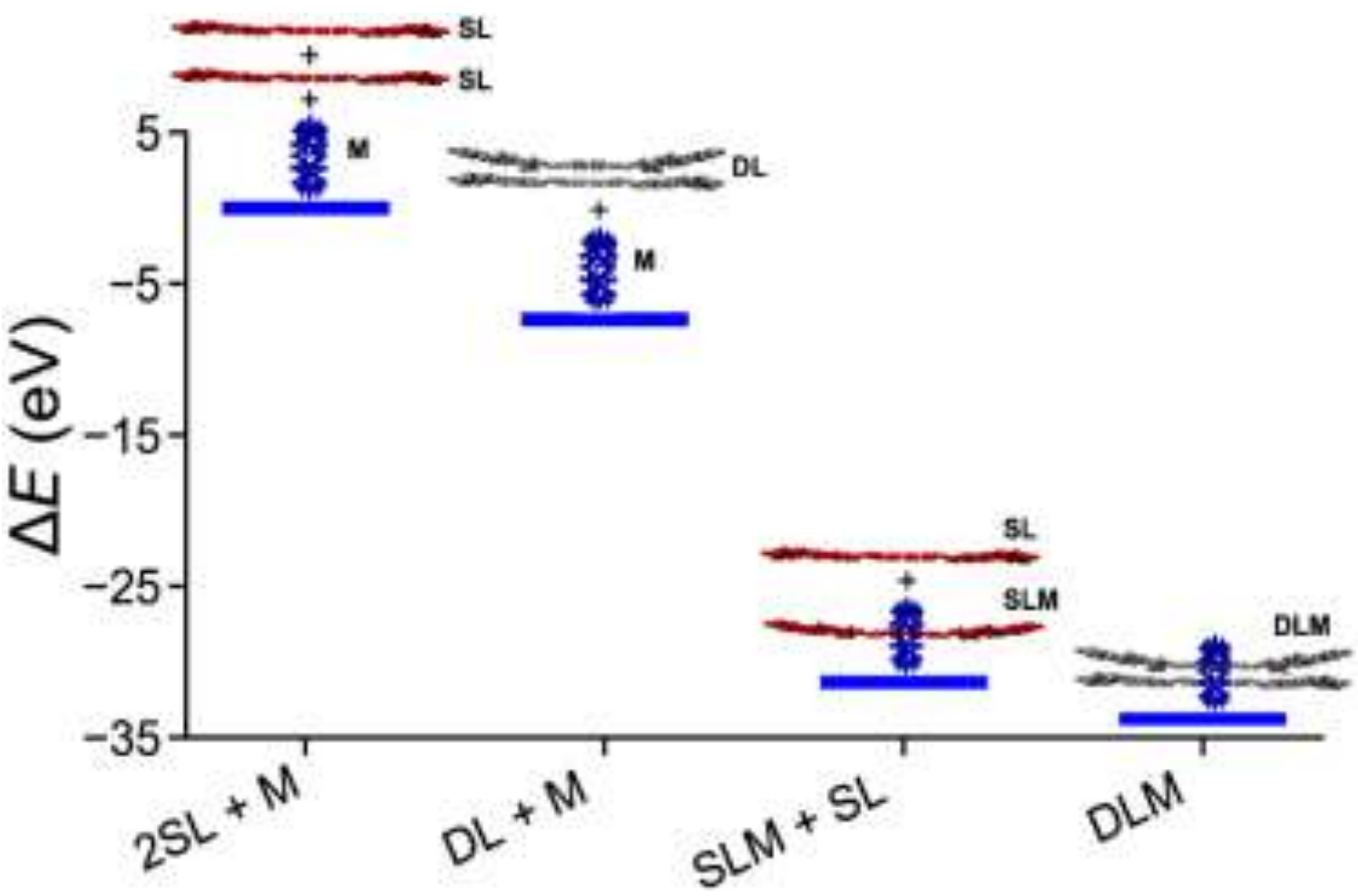

Figure S11: Formation energies (ΔE, eV) for macrocycle incorporation into single-layer and double-layer COF systems. SL denotes an isolated single COF layer, DL denotes an isolated double-layer COF, and M denotes the free macrocycle. SLM denotes a single COF layer with an interlocked macrocycle, and DLM denotes a double-layer COF with an interlocked macrocycle. The reference states are 2SL + M (two isolated single layers and one free macrocycle) and DL + M (one isolated double layer and one free macrocycle). The assembled states are SLM + SL and DLM. Negative ΔE values indicate thermodynamically favorable incorporation. The SLM + SL pathway yields the more negative formation energy, whereas DLM is the lowest-energy assembled configuration.

Thermal stability analysis of VCB: We run three independent simulations of 50 ps at T=650 K, (consistent with the experimental decomposition regime), and at 1000-2000 K. The T=650 K simulations do not show an irreversible decomposition at the given timescale; however, we do observe reversible bond breakings during these simulations. We define bond breaking when the bond length exceeds the bond length by about 20%. We monitor the number of times a particular bond exceeds the threshold (Figure S12a) and the mean first passage time a bond exceeds the threshold (Figure S12b). Results indicate COF C-C bond breaks more frequently, whereas the CB[8] macrocycle exhibits fewer bond-breaking events, primarily involving C–N bonds.

While irreversible bond breakage was not possible for T<2000 K at the simulation time, the 2000 K shows decomposition on the C-C bonds of the COF, consistent with the T=650 K data. Although this temperature is higher than experimental conditions, it provides a qualitative insight of the bond strengths between the linker region and the macrocycle (Figure S12c). Based on this, we conclude that CB[8] macrocycle disruption is not the primary degradation pathway, supporting the experimental thermogravimetric analysis.

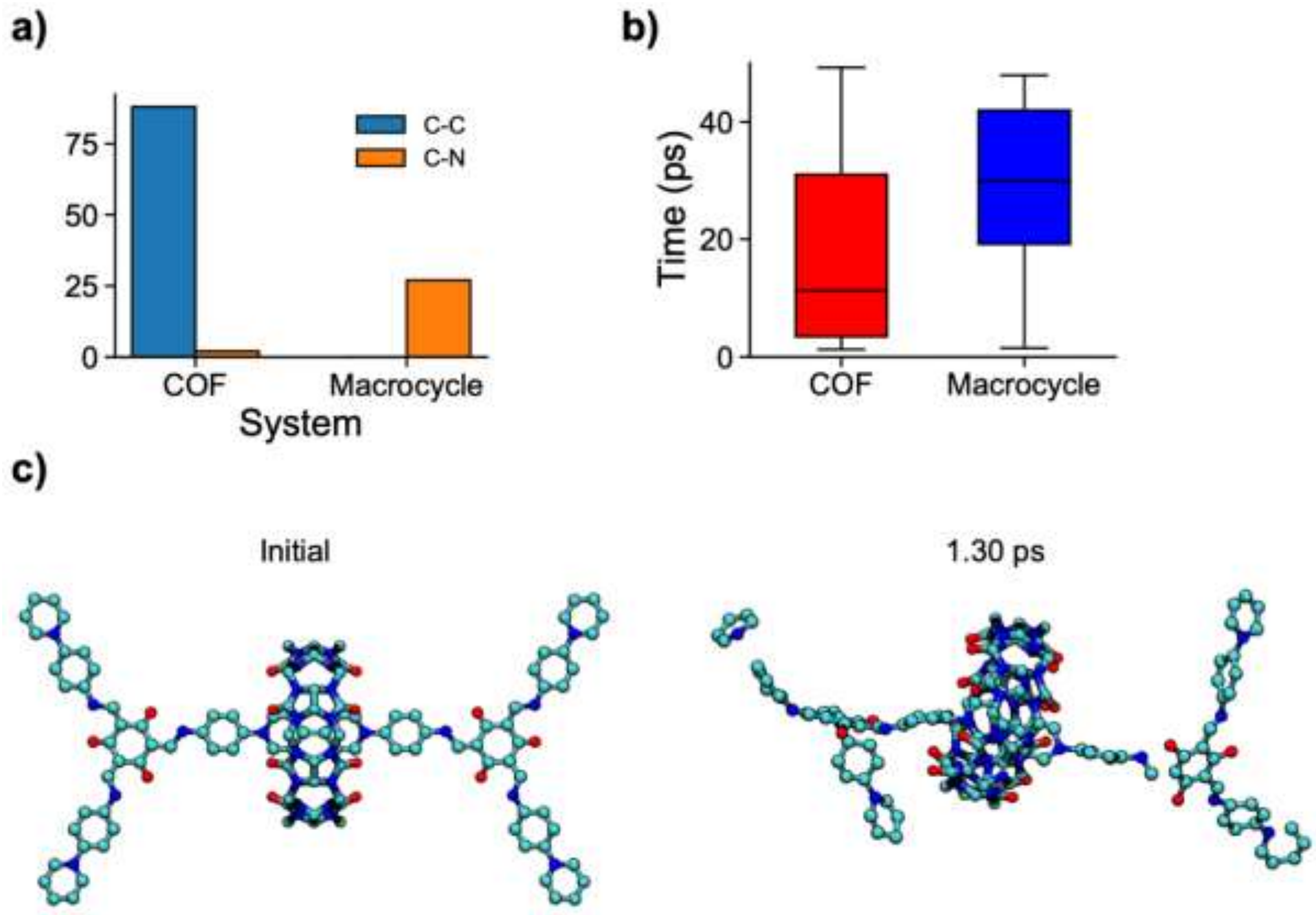

Figure S12: Quantum-based molecular dynamics (QMD) analysis of the thermal stability of representative COF and CB[8] macrocycle fragments. (a) Number of bond-breaking events observed during simulations at 650 K, classified according to C−C and C−N bond types. A bond-breaking event was defined as a bond-length elongation exceeding 20% of its equilibrium value. The COF exhibits more frequent C−C bond-breaking events, whereas the macrocycle displays fewer events, primarily involving C−N bonds. (b) Distribution of bond-breaking times at 650 K. Bond-breaking events occur earlier and over a broader time range in the COF, while the macrocycle exhibits delayed and more localized events, consistent with greater kinetic stability. (c) Representative snapshots from the 2000 K simulation showing the initial structure and the configuration after 1.30 ps. Irreversible bond cleavage is observed within the COF backbone prior to disruption of the CB[8] macrocycle, indicating that degradation is initiated in the framework rather than in the macrocycle under extreme conditions.

Porosity and surface area analysis

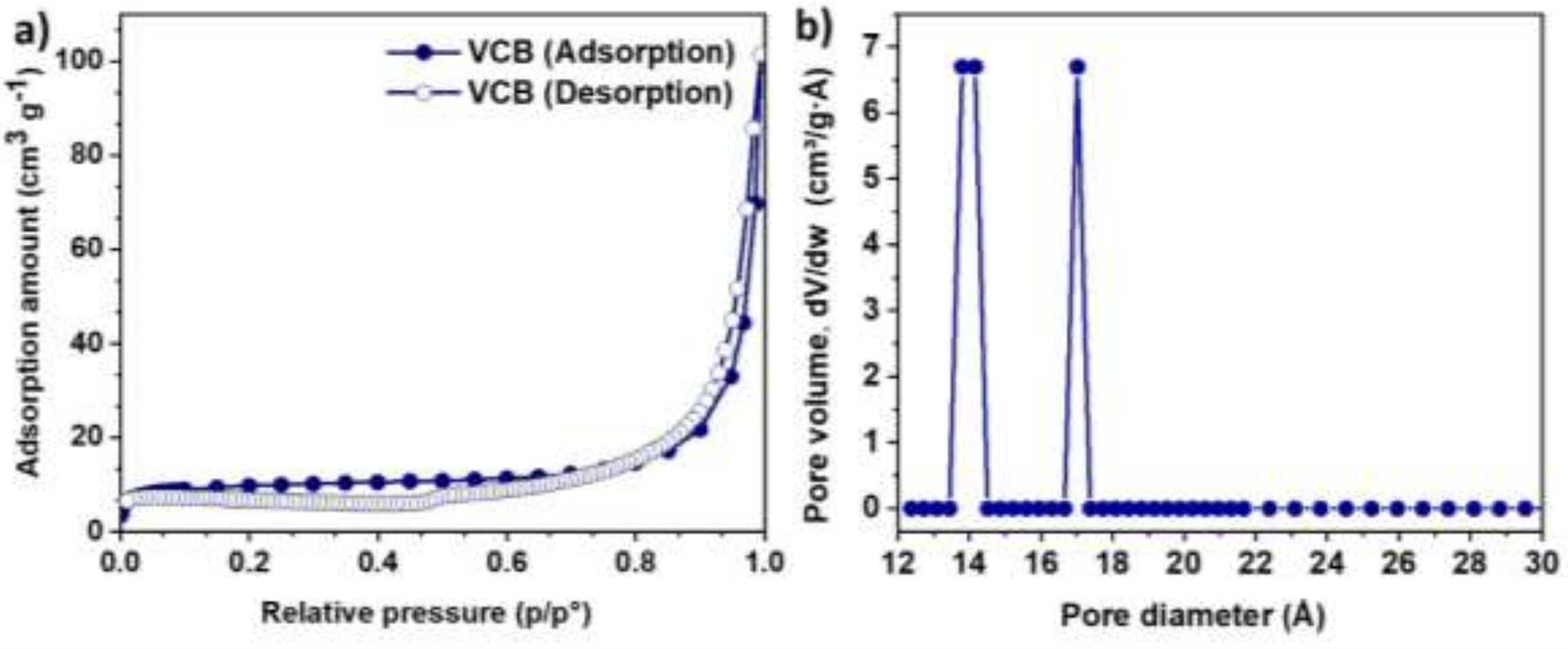

Figure S13: a) N_2 adsorption/desorption isotherm and b) pore distribution of VCB. The slight for the desorption branch to fall marginally below the adsorption branch in the low relative pressure region (P/P_0 = 0-0.6) arises from the extremely low nitrogen uptake and low accessible surface area of the material, which approach the sensitivity limits of the adsorption instrument.

AFM scans and marking for mechanical properties

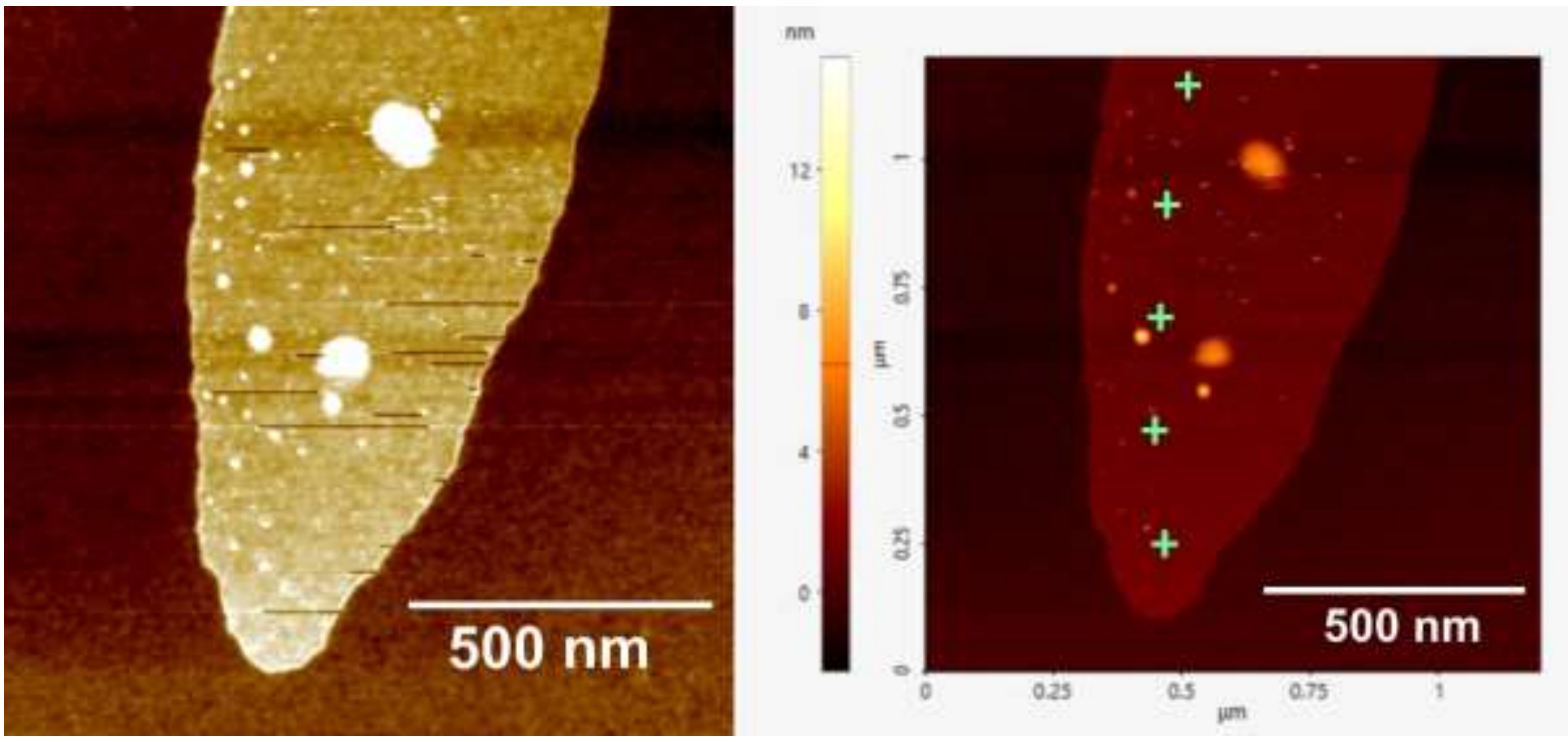

Figure S14: AFM topography was collected prior to the marking of the mechanical property in VCB nanosheets. Five different locations were marked (marked with + sign) and investigated for mechanical property.

Table S1: Mechanical properties comparison for organic materials

COF	Hardness (Mpa)	Modulus (GPa)	Adhesion Energy (fJ)	Pull of force (nN)	Reference
TpV	1.6 ± 0.5	1.9 ± 0.3	50 ± 10	2.7 ± 0.5	21
TpVCB[7]	2.6 ± 0.5	3.0 ± 0.3	80 ± 10	6.0 ± 0.5	21
TABP-PDA	-	1.4	-	-	22
TTA-DHTA	-	25.9 ± 0.6	-	-	23
TABP-BCTA-MCOF	-	0.8 ± 0.3	-	-	24
PPDA-BTCA-MCOF	-	0.5 ± 0.2	-	-	24
TABP-PDA-MCOF	-	0.4 ± 0.2	-	-	24
2D COF-1	-	56.7 ± 7.4	-	-	25
2D polyaramid	-	12.7 ± 3.8	-	-	26
VCB	5.3 ±0.5	4.2 ±0.3	130 ±10	8.7 ±0.5	This work

EPR spectrum of VCB COF with SDT

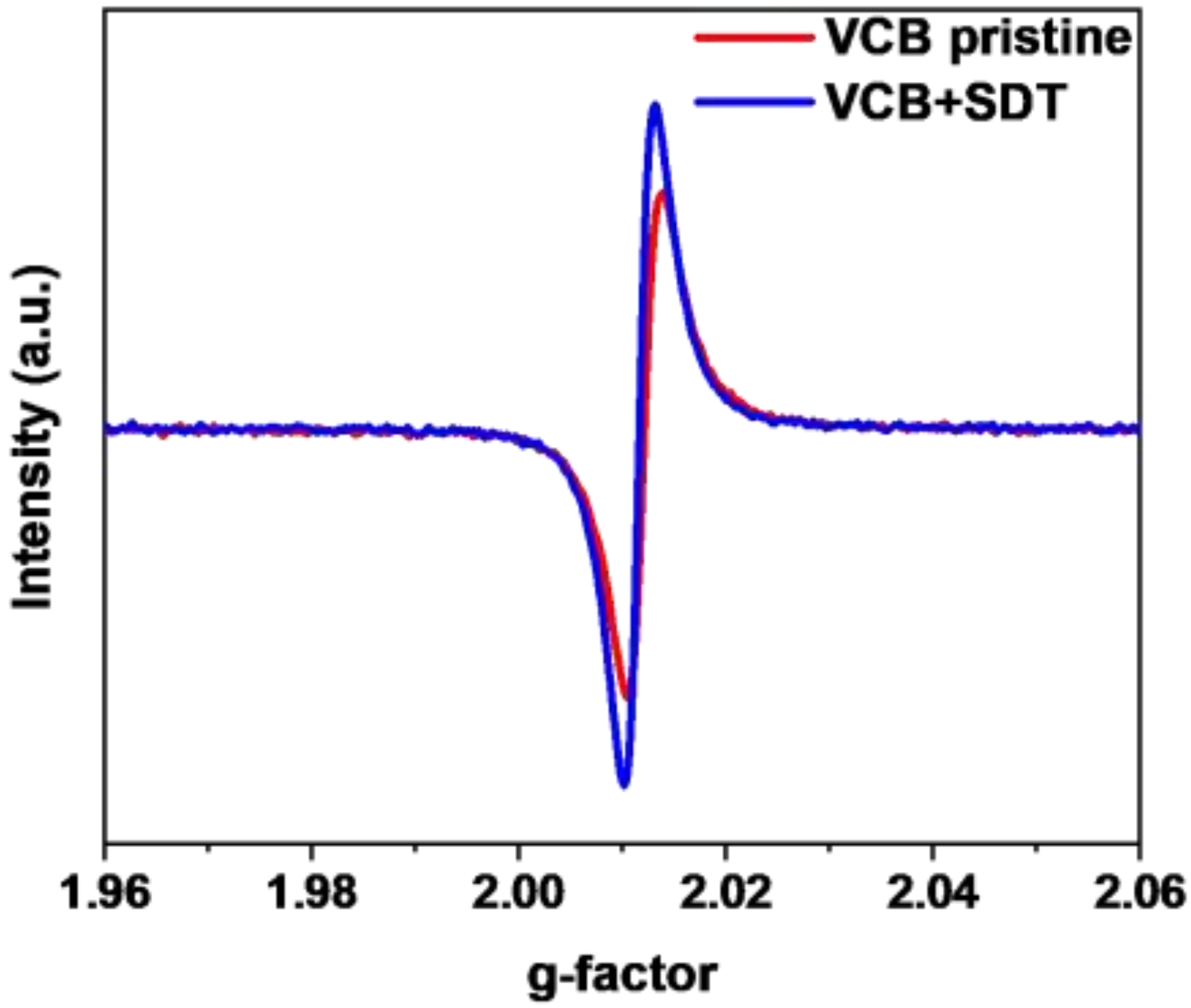

Figure S15: EPR signal of VCB before (red spectrum) and after (blue spectrum) the addition of 1 molar equivalent of sodium dithionite (SDT).

EPR spectrum of VCB and reduced TpV through exposure to air

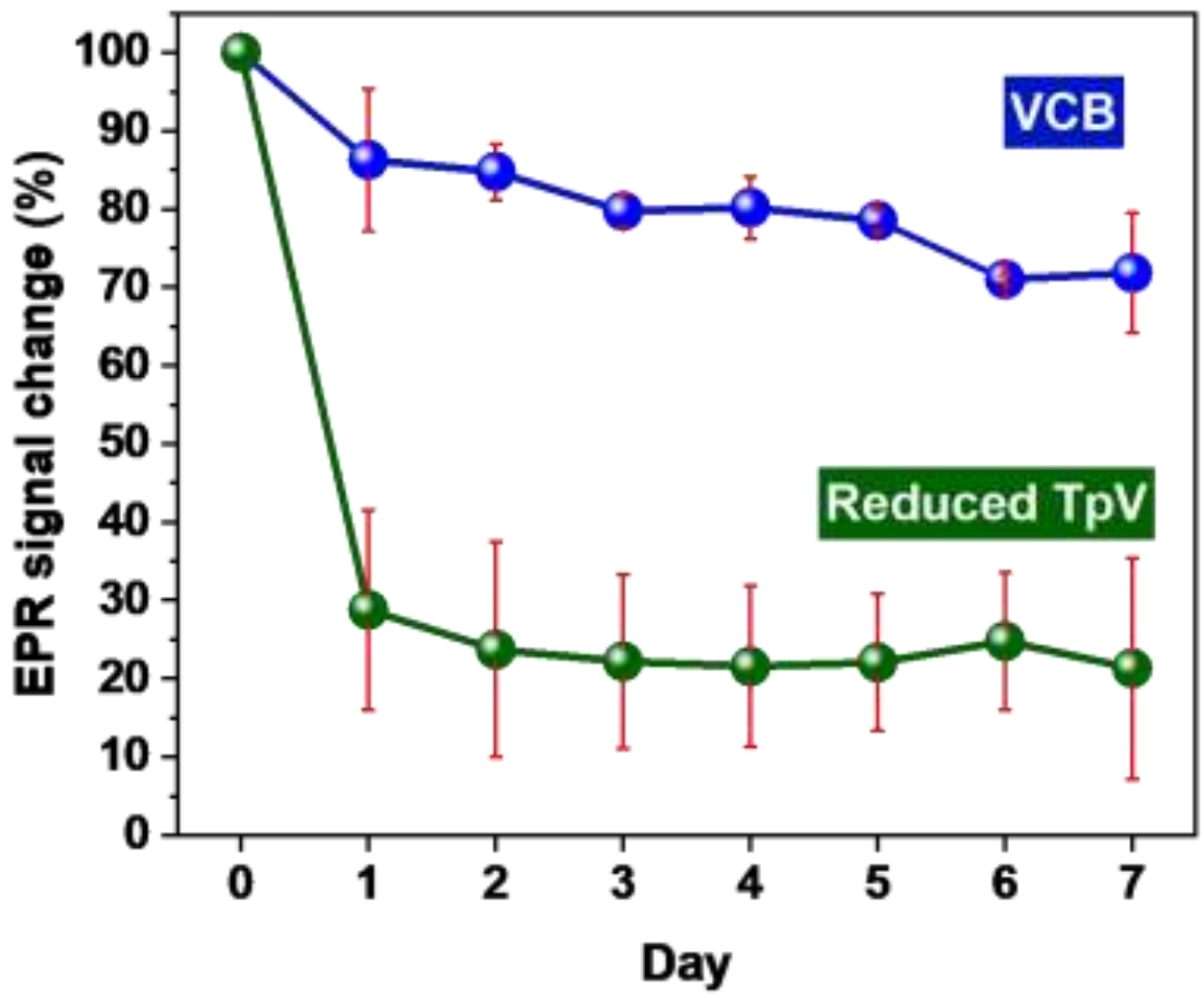

Figure S16: EPR signal of VCB (blue) and reduced TpV (green) during exposure to air for 7 days, showing an overall 28% and 80% decrease in the signal intensity for VCB and reduced TpV, respectively.

EPR and FT-IR spectrum of reduced TpV after CB[8] adsorption and when exposed to air

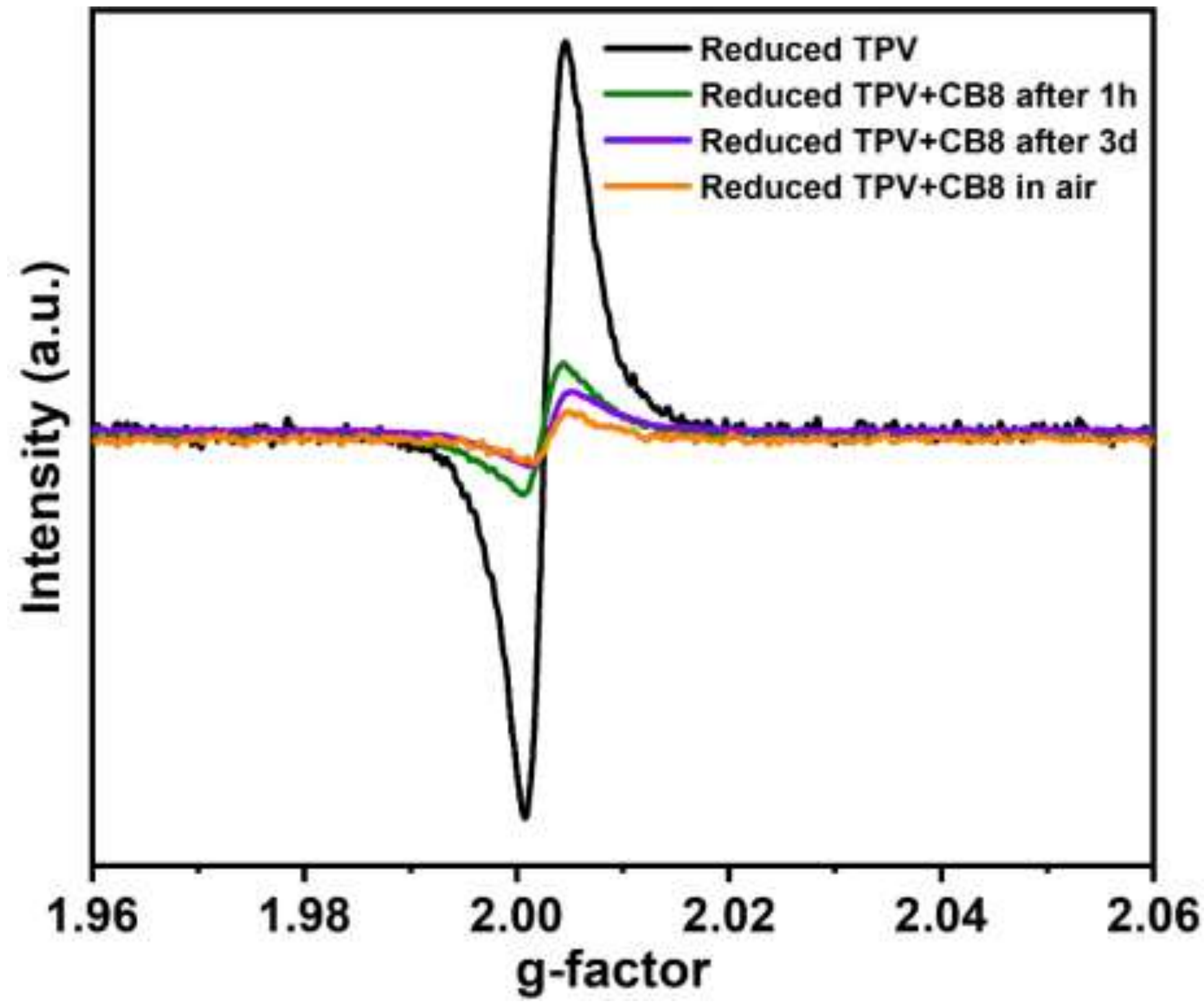

Figure S17: EPR signal of reduced TpV before (black) and after CB[8] adsorption (green and purple) and when the CB[8] adsorbed material was exposed to air (orange).

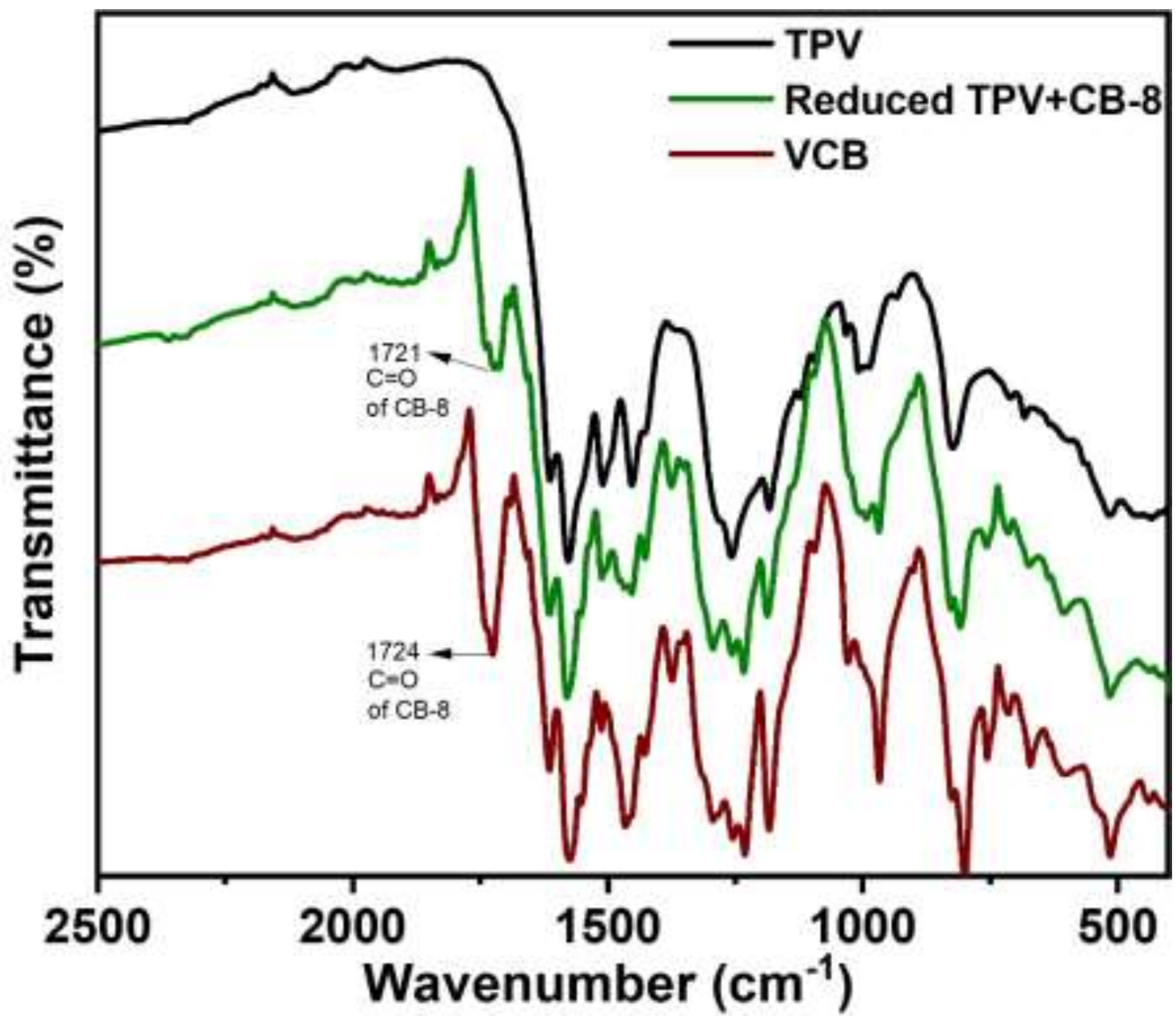

Figure S18: FT-IR spectra confirming the formation of TpV (black) and the adsorption of CB[8] after 3 days soaking (green), matching the FT-IR of VCB (maroon).

Photocurrent density versus time

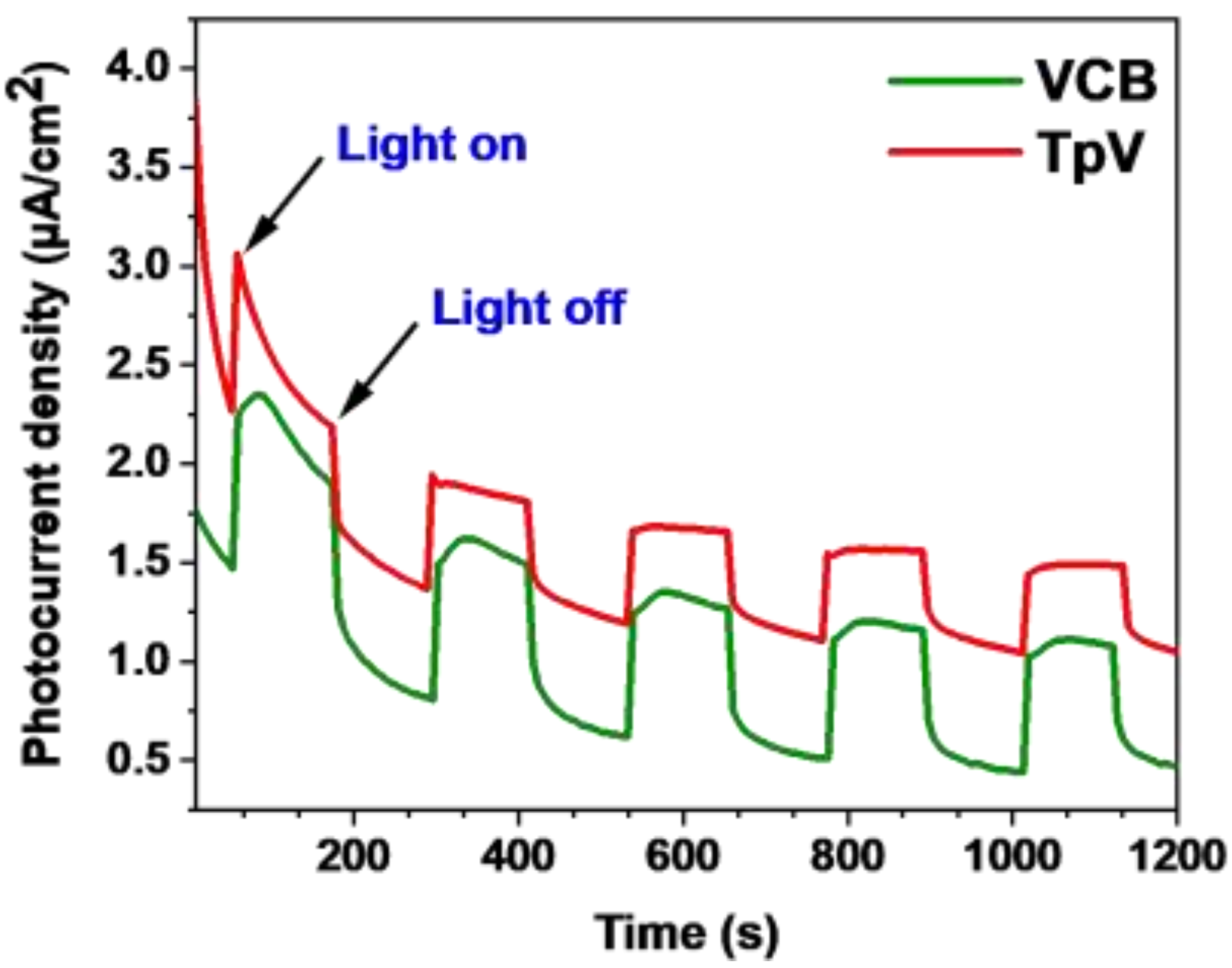

Figure S19: Current density versus time recorded for VCB and TpV for various dark/light cycles (light source: LED 405 nm).

Photoluminescence of VCB and TpV

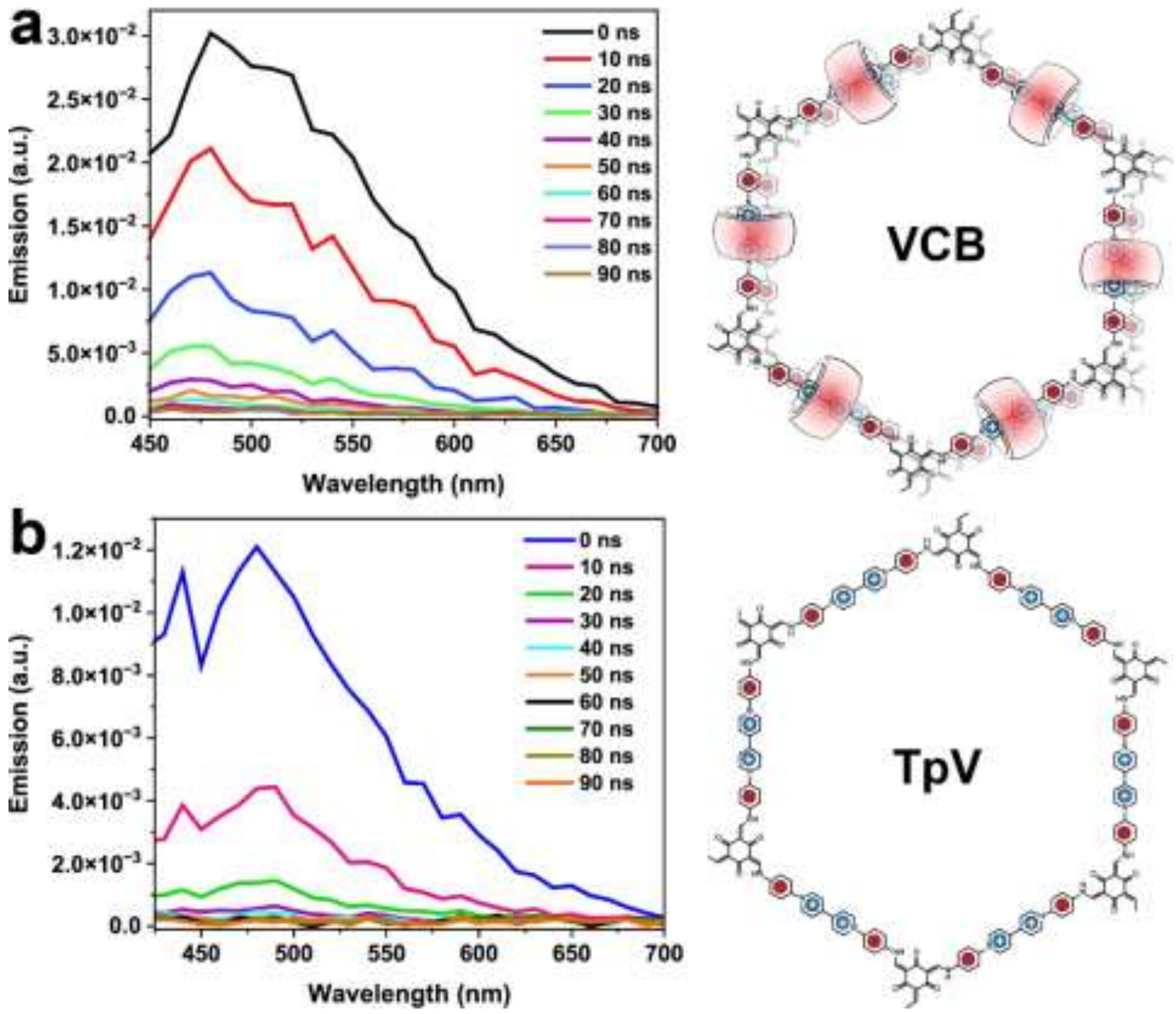

Figure S20: Evolution of the Photoluminescence of a) VCB and b) TpV after excitation at 355 nm (10 ns / spectrum).

REFERENCES

(1) Skorjanc, T., et al., Redox-Responsive Covalent Organic Nanosheets from Viologens and Calix[4]arene for Iodine and Toxic Dye Capture. *Chem-Eur J.* **2018**, 24(34), 8648-8655.

(2) Bi, J.J., et al., Polymer-supported palladium (II) containing N_2O_2: An efficient and robust heterogeneous catalyst for C-C coupling reactions. *J. Polym. Sci. Pol. Chem.* **2018**, 56(20), 2344-2353.

(3) He, L.W., et al., Mechanism unravelling for ultrafast and selective ($^{99}TcO_4^-$) uptake by a radiation-resistant cationic covalent organic framework: a combined radiological experiment and molecular dynamics simulation study. *Chem. Sci.* **2019**, 10(15), 4293-4305.

(4) Bertani, P., et. al., ^{15}N chemical shift referencing in solid state NMR. *Solid State Nucl Mag.* **2014**, 61-62, 15-18.

(5) Thompson, A.P., et al., LAMMPS-a flexible simulation tool for particle-based materials modeling at the atomic, meso, and continuum scales. *Comput. Phys. Commun.* **2022**, 271, 108171.

(6) Mayo, S.L., et. al., Goddard, Dreiding - a Generic Force-Field for Molecular Simulations. *J Phys Chem-Us.* **1990**, 94(26), 8897-8909.

(7) Garai, B., et al., Taming the Topology of Calix[4]arene-Based 2D Covalent Organic Frameworks: Interpenetrated vs Noninterpenetrated Frameworks and Their Selective Removal of Cationic Dyes. *J. Am. Chem. Soc.* **2021**, 143(9), 3407-3415.

(8) Skorjanc, T., et al., Covalent Organic Framework Based on Azacalix[4]arene for the Efficient Capture of Dialysis Waste Products. *ACS Appl. Mater. Inter.* **2022**, 14(34), 39293-39298.

(9) Das, G., et al., Light-driven self-assembly of spiropyran-functionalized covalent organic framework. *Nat. Commun.* **2023**, 14(1), 3765.

(10) Darden, T., et. al., Particle Mesh Ewald - an N.Log(N) Method for Ewald Sums in Large Systems. *J. Chem. Phys.* **1993**, 98(12), 10089-10092.

(11) Valiev, M., et al., NWChem: A comprehensive and scalable open-source solution for large scale molecular simulations. *Comput. Phys. Commun.* **2010**, 181(9), 1477-1489.

(12) Clark, S. J. et al., First principles methods using CASTEP", *Zeitschrift fuer Kristallographie*, **2005**, *220* (5-6), 567-570.

(13) Perdew, J. P., et al., Generalized Gradient Approximation Made Simple. *Phys. Rev. Lett.* **1996**, *77*(18), 3865–3868.

(14) Fletcher, R. (1987) Practical Methods of Optimization. 2nd Edition, John Wiley and Sons, Chichester.

(15) Bannwarth, C., et al., Extended tight-binding quantum chemistry methods, WIREs *Comput. Mol. Sci.* **2020**, *11*, e01493.

(16) Grimme, S. et al., A Robust and Accurate Tight-Binding Quantum Chemical Method for Structures, Vibrational Frequencies, and Noncovalent Interactions of Large Molecular Systems Parametrized for All spd-Block Elements (Z = 1–86). *J. Chem. Theory Comput.* **2017**, *13*, 1989-2009.

(17) Bannwarth, C. et al., GFN2-xTB—An Accurate and Broadly Parametrized Self-Consistent Tight-Binding Quantum Chemical Method with Multipole Electrostatics and Density-Dependent Dispersion Contributions. *J. Chem. Theory Comput.* **2019**, *15*, 1652-1671.

(18) Koopman, J., et al., Calculation of Electron Ionization Mass Spectra with Semiempirical GFNn-xTB Methods. *ACS Omega* **2019** *4* (12), 15120-15133.

(19) Katbashev, A., et al., Overview on Building Blocks and Applications of Efficient and Robust Extended Tight Binding. *J. Phys. Chem. A* **2025** *129* (10), 2667-2682.

(20) Spicher, S., et al., Robust Atomistic Modeling of Materials, Organometallic, and Biochemical Systems. *Angew. Chem. Int. Ed.* **2020**, *59*, 15665.

(21) Das, G., et al., A polyrotaxanated covalent organic network based on viologen and cucurbit[7]uril. *Commun. Chem.* **2019**, *2*, 106.

(22) Zhu, D., et al., Patterning, Transfer, and Tensile Testing of Covalent Organic Framework Films with Nanoscale Thickness. *Chem. Mater.* **2021**, *33*, 6724-6730.

(23) Hao, Q., et al., Confined Synthesis of Two-Dimensional Covalent Organic Framework Thin Films within Superspreading Water Layer. *J. Am. Chem. Soc.* **2018**, *140*, 12152-12158.

(24) Martín-Illán, J. Á., et al., Ultralarge Free-Standing Imine-Based Covalent Organic Framework Membranes Fabricated via Compression. *Adv. Sci.* **2022**, *9*, 2104643.

(25) Yang, Y., et al., Elastic films of single-crystal two-dimensional covalent organic frameworks. *Nature*, **2024**, *630*, 878-883.

(26) Zeng, Y. et al. Irreversible synthesis of an ultrastrong two-dimensional polymeric material. *Nature,* **2022**, *602*, 91-95.
